# Mechanistic Elucidation of Nanomaterial-Enhanced First-Generation Biosensors Using Probe Voltammetry of an Enzymatic Reaction

**DOI:** 10.3390/bios13080798

**Published:** 2023-08-09

**Authors:** Ann H. Wemple, Jamie S. Kaplan, Michael C. Leopold

**Affiliations:** Department of Chemistry, Gottwald Center for the Sciences, University of Richmond, Richmond, VA 23173, USA; holly.wemple@richmond.edu (A.H.W.); jamie.kaplan@richmond.edu (J.S.K.)

**Keywords:** first-generation biosensor, enzyme biosensors, xerogel, nanomaterials, monolayer-protected clusters, carbon nanotube, layer-by-layer assembly, mechanism

## Abstract

The incorporation of nanomaterials (NMs) into biosensing schemes is a well-established strategy for gaining signal enhancement. With electrochemical biosensors, the enhanced performance achieved from using NMs is often attributed to the specific physical properties of the chosen nanocomponents, such as their high electronic conductivity, size-dependent functionality, and/or higher effective surface-to-volume ratios. First generation amperometric biosensing schemes, typically utilizing NMs in conjunction with immobilized enzyme and semi-permeable membranes, can possess complex sensing mechanisms that are difficult to study and challenging to understand beyond the observable signal enhancement. This study shows the use of an enzymatic reaction between xanthine (XAN) and xanthine oxidase (XOx), involving multiple electroactive species, as an electrochemical redox probe tool for ascertaining mechanistic information at and within the modified electrodes used as biosensors. Redox probing using components of this enzymatic reaction are demonstrated on two oft-employed biosensing approaches and commonly used NMs for modified electrodes: gold nanoparticle doped films and carbon nanotube interfaces. In both situations, the XAN metabolism voltammetry allows for a greater understanding of the functionality of the semipermeable membranes, the role of the NMs, and how the interplay between the two components creates signal enhancement.

## 1. Introduction

For decades now, electrochemical biosensor research has enabled the development of practical and effective sensors for the detection and monitoring of target species relevant to clinical, environmental, and industrial applications with many recent reviews available to collectively summarize more recent progress and the current challenges of the field [1,2,3,4]. Even after years of development, many traditional strategies, materials, and schemes are still widely employed, such as a transducer (i.e., an electrode) typically modified with biomolecules and semi-permeable membranes to provide sensor selectivity and measurable current response in the presence of low concentrations of targeted analytes (sensitivity) [5]. A popular strategy within biosensor research concerns amperometric sensors that utilize the natural selectivity of immobilized enzymes to directly detect their corresponding substrate or, as in the case of first-generation amperometric biosensors, indirectly detect another target molecule through an enzymatic reaction [6,7,8,9,10,11]. Within that realm, a common approach aimed at improving biosensor sensitivity is the incorporation of nanomaterials (NMs), which can range from metallic nanoparticles (NPs) or inorganic NPs to carbon-based NPs, such as carbon nanotubes (CNTs), as functional components of the designed sensing schemes [1,12,13,14,15,16,17]. 

The motivations of biosensor research vary greatly and often can revolve around achieving some high value sensing attribute such as the point-of-care detection of a medically relevant target [18,19,20,21], highly mobile or wearable devices [22,23], highly flexible transducers [24] (e.g., paper- [25] or sticker-based [26] electrodes), or the miniaturization of sensing schemes to microneedles for the potential of *in-vivo* [27] or transdermal operation [28]. Research in this area can also be highly application-focused, with literature reports describing electrochemical biosensors for monitoring food quality [29], detecting environmental contaminants (e.g., heavy metals, pesticides) [3,30], or reporting the presence of narcotics [31]. While many of the reports in the literature use NMs to achieve their sensing goals, the exact role of the incorporated materials is often not described in detail and signal enhancement is usually simply attributed to the physical properties (e.g., surface-to-area vs. volume ratio) of the NMs. Other than the observed signal-to-noise (S/N) improvement when using NMs, actual sensing mechanisms are often difficult to ascertain or systematically study. 

**Figure 1:**
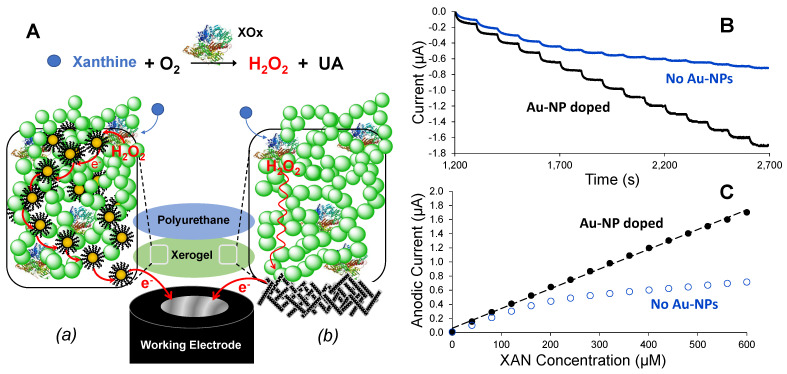
(**A**) Schematic illustrations of two different xanthine (XAN) biosensing schemes employing xerogel scaffolds for immobilized xanthine oxidase enzyme (XOx), polyurethane-based semi-permeable membranes for selectivity, and strategically incorporated NMs: (**a**) gold NP-doped xerogels and (**b**) surface modification of the working electrode interface with CNTs. In either scenario, the inclusion of NMs typically results in signal enhancement that subsequently leads to more sensitive biosensing, faster response times, and extended linear ranges, as shown by the illustrative example data of (**B**) amperometric I-t and (**C**) corresponding calibration curves showing the effect with and without Au-NP incorporation (**A**(**a**)—**left**) for xanthine biosensing. Note: Similar signal enhancements from the CNT scheme (**A**(**b**)—**right**) were also demonstrated for XAN (Supporting Information, Figure S1).

Through prior work in our lab, we have studied various iterations of NM-enhanced, first-generation amperometric biosensors that employ immobilized enzymes to catalyze reactions between a substrate molecule and oxygen to produce H_2_O_2_, an enzymatic reaction by-product subsequently oxidized at the electrode interface that generates an anodic current (i.e., signal), indicating the presence of a targeted molecule (i.e., an “indirect” biosensor). The development and mechanistic understanding of first-generation biosensing schemes constructed with layer-by-layer (LbL) methodology and augmented with NMs remains our primary interest. One of the major materials utilized as a scaffold for both enzymes and NMs at the electrode interfaces are xerogels: porous, silane-based, polymeric films that are often complemented with the use of various semi-permeable membranes [32,33]. Using these materials and different assembly strategies, we have successfully developed biosensors for a range of target molecules with both clinical and/or industrial relevance, including schemes for the detection of glucose (diabetes) [34], uric acid (pre-eclampsia) [33], sarcosine and creatinine (prostate cancer) [35], galactose (galactosemia) [36], lactate (sepsis) [27], and xanthine (urinary track disease, Lesch-Nyhan Syndrome, and/or meat freshness) [37]. Similar to other work in this area [1,12,13,14,15,16,17,38], the vast majority of biosensors developed in our lab also employ various NMs to improve S/N and, in virtually every case, utilize additional membrane layers for added selectivity (e.g., polyurethane [34] and chitosan [35], for example). Figure 1 presents two generic LbL-constructed biosensing schemes applied to xanthine (XAN) detection that feature two different NMs in established schemes that result in signal enhancement [34,36]. The signal enhancement from inclusion of Au-NPs is evident in both the collected amperometric responses and corresponding calibration curves with and without inclusion of Au-NPs (Figure 1B,C—*right*). A biosensor utilizing such a scheme showed promising results in detecting XAN levels above normal in bodily fluids (>4 µM in serum; >160 µM in urine)—a useful early diagnostic tool for disease [37]. In a demonstration of the versatility of materials and strategy, similar materials were employed in a CNT-based biosensing scheme (Figure 1A(b)) that was readily adapted to XAN detection (previously unreported) to show similar signal enhancement effects (Supporting Information, Figure S1). Both of these approaches have been successfully executed in the development of a number of different biosensors where the analytical performance and figures of merit have been carefully measured and compared to other types of schemes [27,34,35,36,37,39]. For the purpose of the current mechanistic study, however, these two different approaches illustrate two of the major strategies for how NMs are typically incorporated into biosensing schemes: (1) the inclusion of NMs within an entire layer of the scheme (Figure 1A(a)) or simply modifying the working electrode directly with NMs (Figure 1A(b)). As in the other biosensing literature of this nature, the role and relevance of the NMs as a functional component of the biosensor’s mechanism are often unaddressed in detail because it is challenging to measure and/or observe. 

In this study, a xanthine (XAN) biosensing scheme involving an enzyme metabolism was employed as a probe reaction to systematically study and understand the sensing mechanisms and signal enhancement capabilities of these NM-enhanced, first-generation biosensing schemes. Using the unique aspects of the enzymatic reaction of XAN with xanthine oxidase (XOx) as an effective redox probe, we specifically target xerogel-based biosensing schemes that incorporate either networks of gold NPs [27,34,37,39] or systems involving films infused with CNTs at the electrode interface [35,36]. Systematic studies using sweep voltammetry and amperometric measurements reveal that NM-based signal enhancement is critically dependent on NM placement and their interplay with the semi-permeable membranes. This study finds that both the materials, working in concert, fundamentally change the interfacial electrochemistry to produce an enhanced signal. Given the prevalence of these materials in numerous biosensing schemes, the demonstrated voltammetry tool may be able to provide greater mechanistic understanding about the functionality of components within a range of layered biosensing schemes and should be of significant interest to the field. 

## 2. Materials and Methods

### 2.1. Reagents and Instrumentation

All chemicals and reagents were purchased in high grade from Millipore-Sigma (St. Louis, MO, USA) or Oakwood Chemical (Columbia, SC, USA). Hydroxy-methyltriethoxysilane (HMTES) was purchased from Gelest Incorporated (Morrisville, PA, USA), Tecoflex polyurethane ((SG-80A)) was purchased from the Lubrizol Corporation (Cleveland, OH, USA), while Hydrothane polyurethane (AL25-80A) was obtained from AdvanSource Biomaterials (Wilmington, MA, USA). Xanthine oxidase enzyme (XOx) was purchased from Creative Enzymes (Shirley, NY, USA), while catalase (CAT) was ordered from either Millipore-Sigma. The gold NPs used in this study, hexanethiolate (C6)-protected gold clusters known as monolayer-protected clusters (C6-MPCs), were synthesized in-house according to established procedures and characterized to have an *average* composition and diameter of Au_225_C6_75_ and ~2.0 nm, respectively [37]. Single-walled CNTs with carboxylic acid functional groups were purchased from Nano Lab Inc. (Waltham, MA, USA). Voltammetry and amperometry results were obtained with potentiostats (1000B, 1030C, or 420B models) from CH Instruments (Bee Cave, TX, USA) using Ag/AgCl (saturated KCl) aqueous reference electrode (RE) (CH Instruments) and platinum auxiliary electrode (CE) (Millipore-Sigma). Specific electrochemical parameters for cyclic, differential pulse, and Osteryoung square wave voltammetry (SWV) are provided in the Supporting Information.

### 2.2. Preparation of Biosensor Systems

Both platinum (2 mm diameter) and glassy carbon electrodes (GCE) (3 mm diameter) were polished on a polishing wheel affixed with a polishing cloth pre-treated with successively smaller (1.0, 0.3, and 0.05 μm) suspensions of alumina powders (Buehler, Lake Bluff, IL, USA) and ultra-pure (UP) H_2_O (18.02 MΩ∙cm) as an extender. Both types of electrodes were subsequently excessively rinsed with UP H_2_O and the platinum electrodes then underwent cyclic voltammetry in diluted H_2_SO_4_ (0.1 M) between +1.2 and +0.25 V until voltammograms were consistent with clean platinum surface oxidation/reduction. All electrodes were then rinsed with UP H_2_O and dried in a N_2_ stream before proceeding with modification (below). 

### 2.3. MPC-Doped Xerogel-Biosensing Schemes

For xerogel formation and electrode modification featuring MPCs, previously developed procedures were generally followed [37,39]. Briefly, a deposition mixture formed with 50 µL of HMTES in EtOH (50% *v*/*v*) (stored in a desiccated glovebox and transferred to a microcentrifugation tube under controlled atmosphere) was mixed with 200-proof ethanol (50% *v*/*v*), so that the final ratio of ethanol to HMTES was 75:25 *v*/*v* before THF (75 µL) was added to the mixture. For xerogel systems featuring C6-MPC doping, MPCs in a ratio of 1:400 to the silane were dissolved in this mixture as well [40]. In a separate vial, 12 mg of XOx enzyme was dissolved in 75 μL of UP H_2_O. Both vials were vortexed for complete mixing prior to 50 μL of the aqueous XOx being transferred to HMTES/THF (C6-MPC) mixture tube that was subsequently mixed thoroughly until homogenized. An aliquot of this mixture (3 μL) was drop-cast directly onto the clean electrode and then placed inside a humidity chamber set to 50% relative humidity (RH) for 48 h of drying/aging (cross-polymerization and formation of xerogel). After 48 h of aging, the HMTES xerogel-modified electrodes were often treated with a semi-permeable blended polyurethane (PU) outer layer, previously described and characterized in other biosensor studies by our lab and others [32,33,37]. Briefly, an xanthine optimized [37] blend of 75% HPU (e.g., 75 mg) and 25% TPU (e.g., 25 mg) dissolved in 5 mL of THF/EtOH (50% *v*/*v*) was stirred overnight and sonicated for complete dissolution. Portions (10 µL) of the PU solution were drop-cast on top of the XOx embedded and/or C6-MPC doped xerogels and allowed to dry in the humidity chamber (30 min). As in other studies using these materials [27,37], assembled biosensors were soaked in 10 mM potassium phosphate buffer (PBS; pH 7.0) for an hour prior to use as to ensure hydration and equilibrium of the PU and xerogel layers [37]. 

### 2.4. Xerogel-Biosensing Schemes with CNTs

Previously established procedures [36] were followed for the formation modified electrodes featuring single-walled carbon nanotubes with carboxylic acid functionalization (SWCNT-COOH) with XOx-doped HMTES xerogels and PU capping layers [36,37]. SWCNTs-COOH were dissolved in a 1% Nafion solution at 1 mg/mL before a 5 µL aliquot of the solution was applied to a clean electrode and allowed to dry at ambient conditions (~30 min). HMTES sol-gel mixtures and the PU blend were made and deposited as described in the previous section once SWCNT-COOH-modified electrodes were created and dried, including allowing 48 h for HMTES xerogel to form and age (in the humidity chamber (50% RH) prior to the application of the PU blend (30 min; 50% RH). As in other studies using these materials, assembled biosensors were soaked in 10 mM potassium phosphate buffer (PBS; pH 7.0) for an hour prior to use as to ensure hydration and equilibrium of the PU and xerogel layers [37]. 

## 3. Results and Discussion

The voltammetry of solution redox probes is a longstanding electrochemical tool for the assessment of modified electrodes with common probe molecules being potassium ferricyanide and ruthenium hexamine [41]. Redox probe voltammetry is traditionally carried out in the solution at the electrode interface (i.e., external to solution) with the shape and nature of the observed voltammetry serving as an indicator about the surface modification of the substrate. In this study, we take a different approach to the redox probing of interfaces and utilize the products of an enzymatic reaction within a biosensor scheme to ascertain real-time chemistry information, not only at the electrode–solution interface but also within assembled layers of materials that comprise the biosensor. Xanthine (XAN), a critical species in purine metabolism, represents a molecule of interest for significant sensor development research as it is relevant to both clinical and industrial applications. As such, the literature is rich with reports of optical and electrochemical XAN sensors and biosensors [42], including examples of first-generation amperometric biosensors utilizing a xanthine oxidase (XOx)-catalyzed enzymatic reaction to produce detectable H_2_O_2_ at modified electrodes [37,43]. These type of biosensing schemes typically hold the working electrode at a high potentials (oxidative and reductive) during exposure to XAN in order to immediately generate a current (i.e., signal) stemming from the electrochemistry of the enzyme reaction H_2_O_2_ product [44]. Somewhat unique to XOx, as depicted in the Supporting Information (Scheme S1), in comparison to other enzymes, is that, in addition to the XAN substrate, multiple products of its reaction are electroactive (**bold**), including H_2_O_2_ and uric acid (UA), depending on applied potential (E, Volts):

(1) $$XAN+O_{2}\;\overset{XOx}{\to }\;UA+H_{2}O_{2}$$

(2a) $$H_{2}O_{2}\;\to \;{2H}^{+}+O_{2}+2e^{-}$$

(2b) $$UA\;\to \;{UA}^{2+}+2H^{+}+2e^{-}$$

In this study, we will use these electroactive species as both external (solution) and internal (interfacial) redox probes to help understand the mechanistic aspects of NM-enhanced biosensing schemes. 

To establish an electrochemical baseline for these electroactive species, the results of typical sweep voltammetry methods, including cyclic voltammetry (CV), differential pulse voltammetry (DPV), and square wave voltammetry (SWV), carried out on a mixture of XAN, HXAN, and UA at a clean glassy carbon electrode (GCE), are provided in Supporting Information (Figure S2). The voltammograms and peak potentials observed are all consistent with numerous literature reports [45,46] and show asymmetric redox activity, where the oxidation of the compounds is significantly more prominent. As such, many biosensor schemes involving these species often target the oxidation of these compounds. Additionally, it is well known that the voltametric sensitivities of certain electroactive species, particularly H_2_O_2_ electrochemistry at or near physiological pH, can be dependent on the electrode interface (i.e., different metal composition and/or modification), applied potential, and solution conditions (e.g., pH, electrolyte concentration) [33,37,47,48,49,50]. These parameters are especially relevant for first-generation biosensing schemes that rely on H_2_O_2_ electrochemistry. As such, the sensitivities of the redox species involved in XAN metabolism at platinum and GCEs, both very common transducers in biosensing schemes, must be established. 

**Figure 2:**
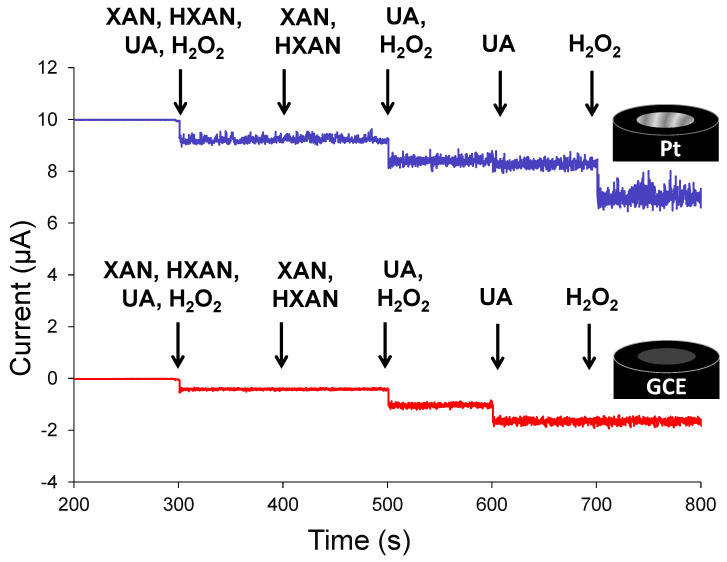
Representative I-t curves for clean (unmodified) platinum (top) and glassy carbon (bottom) electrodes, held at +0.65 V and monitored for oxidative (i.e., anodic) current during standardized 20 µL injections of 10 mM four-component mixtures, two-component mixtures, or individual electroactive species, resulting in 100 µM solutions in 10 mM PBS (pH 7) bulk solution. Note: I-t curve at Pt (top trace) is offset +10 µA for clarity.

Figure 2 shows typical amperometric behaviors, known as current–time (I-t) plots, for platinum and GCEs immersed in potassium phosphate-buffered solution (PBS) and held at a common biosensor oxidizing potential (+0.65 V) during standard injections of either individual components or mixtures of HXAN, XAN, UA, and H_2_O_2_. As expected from preliminary voltammetry provided in the Supporting Information (Figure S1), no oxidative responses during either XAN or HXAN injections were observed in the I-t curves. The results do, however, show that the platinum interface is more sensitive toward H_2_O_2_ oxidation (minimal UA oxidation), while the GCE electrode is more sensitive toward UA oxidation (minimal H_2_O_2_ oxidation) [37]. In this manner, the applied potential and the type of working electrode as well as the knowledge of its corresponding sensitivity toward specific species can all be used to probe biosensing mechanisms. Notably, conducting the same injections in reverse order at each of the interfaces did not change this result, as illustrated in the Supporting Information (Figure S3). Additionally, the cyclic voltammetry of H_2_O_2_ shows voltametric activity, albeit not well-defined, at platinum electrodes (Supporting Information, Figure S4). 

### 3.1. Nanoparticle Network-Enhanced Xerogel Biosensing Schemes

**Figure 3:**
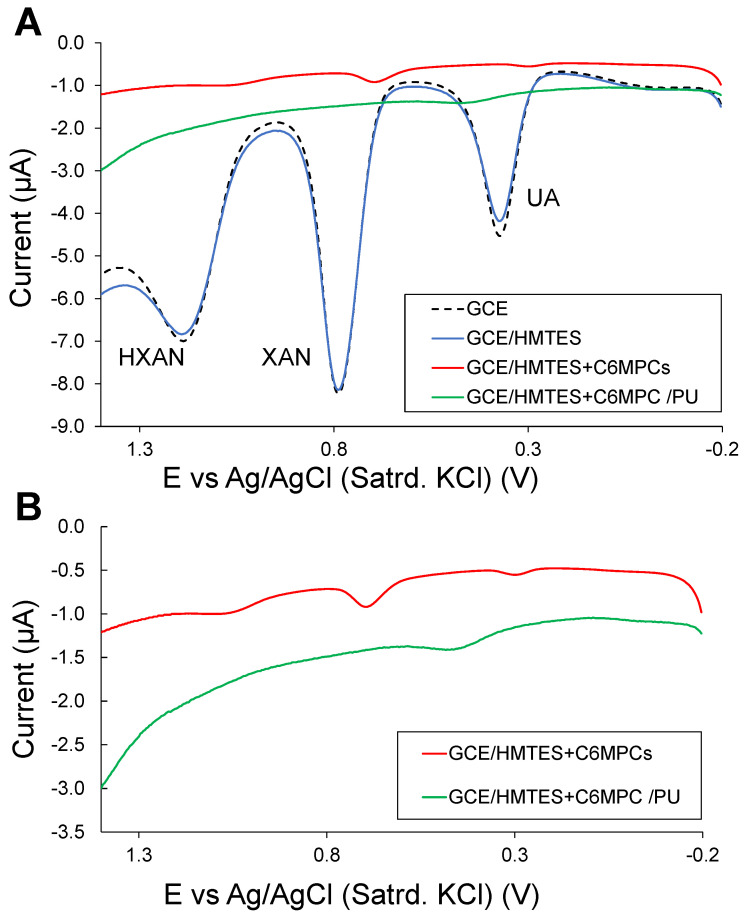
(**A**) Typical DPV results for various modified GCEs immersed in a solution of 0.25 mM mixture of HXAN, XAN, and UA (10 mM PBS; pH 7), illustrating effective blocking behavior toward the diffusional solution electroactive species of MPC-doped HMTES xerogel and PU-capped C6-MPC-doped HMTES xerogels versus un-doped HMTES xerogel at GCE and bare GCEs. (**B**) Isolated DPV results for the MPC-doped systems from (**A**) showing the order-of-magnitude smaller current and lack of clearly defined peaks. Note: XOx is excluded from these solutions to isolate and identify HXAN, XAN, and UA signals.

These LbL-constructed schemes typically involve modifying the electrode with HMTES xerogel that houses the immobilized XOx enzyme and is doped with hexanethiolate-passivated gold NPs, also known as monolayer-protected clusters (MPCs) [51]. The HMTES xerogel is then capped with a layer of blended polyurethane (PU) that was previously used as an effective outer selective membrane in systems and is known to significantly enhance biosensing signals [37]. The proposed hypothesis for the mechanism of said enhancement is that XAN permeates the PU layer along with O_2_ and is then metabolized by XOx into UA and H_2_O_2_ before the products, depending on the applied potential, are oxidized at the MPC-network embedded in the xerogel, which subsequentially allows for fast electronic communication to the electrode interface [40]. The PU blend of 75:25 HPU:TPU was determined to be most optimal for XAN permeability in a previous XAN biosensor report [37]. In order to probe the mechanism of the biosensor for this study, we first constructed the biosensor scheme at GCEs without embedding XOx in the HMTES xerogel layer. This exclusion was to preclude H_2_O_2_ production and subsequent oxidation, thereby allowing for the easy identification and monitoring of other electroactive species that may be present during purine metabolism. Figure 3A shows DPV results for this system in the mixture of HXAN, XAN, and UA at various stages of construction: a bare GCE, an undoped (i.e., no MPCs) HMTES-modified GCE, and a full system (i.e., GCE modified with C6-MPC-doped HMTES) with and without the PU capping layer. This experiment serves to use HXAN, XAN, and UA as the more traditional, external, diffusional electrochemical probes at modified electrodes known to be sensitive toward these species. Notably, the full systems with MPC doping, both with and without the PU capping layer, are shown to be significantly blocking toward all three components of the mixture versus the same species at the undoped HMTES-modified and bare GCEs. Figure 3B shows an expansion of the voltammetry for the two more blocking modified electrodes (full systems with and without the PU capping layer) to illustrate the order-of-magnitude smaller current responses and nearly non-existent voltametric peaks for the components. It can therefore be concluded that the C6 MPC-doped HMTES xerogels, both with and without the PU capping layer, are extremely effective at blocking diffusional species in solution from the electrode.

**Figure 4:**
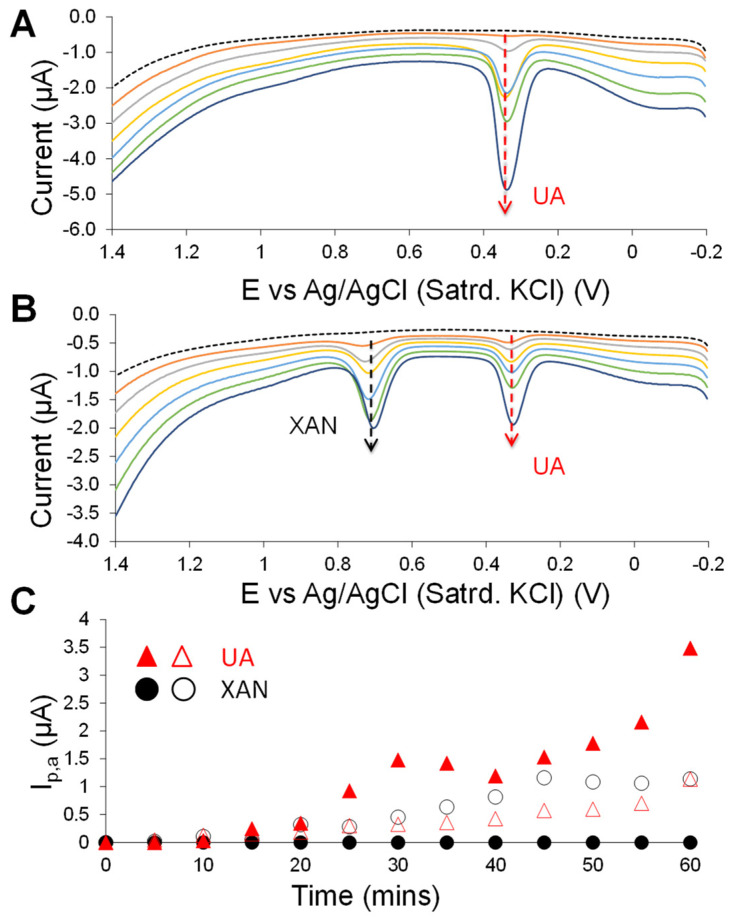
Sequential DPV scans over time before (dashed trace) and after (solid traces) exposure to 200 µM XAN (10 mM PBS; pH 7) at GCEs modified with C6-MPC-doped HMTES xerogels (with XOx) either (**A**) with or (**B**) without the PU capping layer (scans every 10 min shown); (**C**) oxidative, anodic peak currents (I_p,a_) measured from the previous DPV results (**A**,**B**) at +0.380 V for XOx generated UA and +0.725 V from injected XAN at C6-MPC-doped HMTES systems with (solid markers) and without (open markers) PU capping layers measured over the course of 1 h.

The same C6 MPC-doped HMTES-modified GCEs, with and without the PU capping layer but embedded with XOx, were immersed in a quiescent PBS and scanned over time using DPV before and after injection XAN (200 µM) into the solution. As such, the only source of XAN is diffused via the injection, while the only source of UA would be the product of the XOx-catalyzed enzymatic reaction. Figure 4A shows DPV scans before and after (measured every 5 min) injection of XAN into the solution at the PU-capped system. With the PU layer in place, the only signal observed at the electrode interface is the UA peak (+0.4 V), as it is produced within the film, with the “effective” diffusion layer now limited to under the PU layer. The electrochemical signal from the injected XAN diffusion to the capped system is not observed at all. The same system without the PU capping layer produces substantial XAN and UA signals, which subsequently increased over time (Figure 4B). A similar DPV analysis of the system without either the PU or embedded XOx (Supporting Information, Figure S5) shows a signal only for XAN oxidation, increasing over time as it diffuses to the electrode through the layering. Eventually, at longer times (e.g., after 1 h), the magnitude of the current stabilizes and reaches a near steady-state response in these systems (Supporting Information, Figure S6). Figure 4C summarizes the anodic peak current measured at ~+0.380 and +0.725 V for UA and XAN, respectively. As seen in the results, tracking the anodic current generated in these systems (e.g., with and without the PU capping layer) for one hour shows the abruptly increasing UA signal along with virtually no signal from XAN with the PU-capped films (solid symbols).

Both UA and XAN slowly increase at the film without the PU layer (open symbols). These results suggest that the PU layer is a critical component to the sensing mechanism and achieve effective signal enhancement from the inclusion of NMs. The PU serves to effectively “gate” or slow down the entrance of the XAN into film system, avoiding the overwhelming of the XOx enzyme and Michaelis–Menton kinetic effects [40], while simultaneously emphasizing a diffusion layer that effectively exists under the PU layer. 

**Figure 5:**
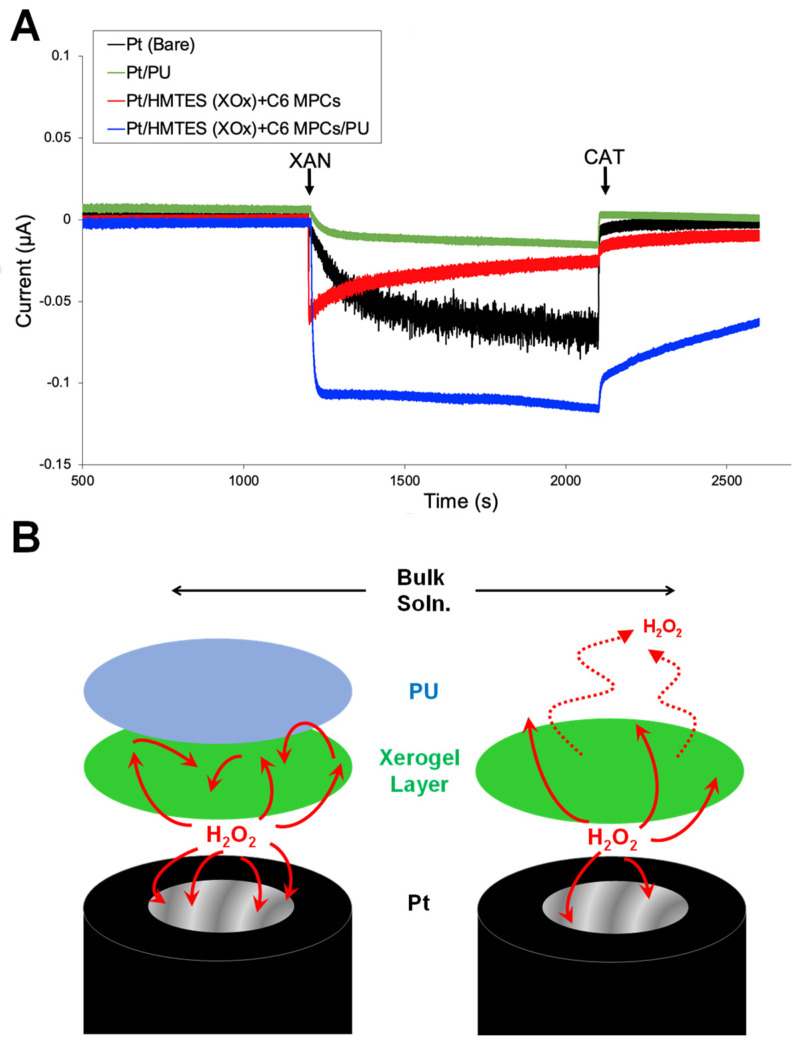
(**A**) Amperometric I-t curves of platinum electrodes in PBS (10 mM; pH 7) held at +0.380 V and modified with C6 MPC-doped HMTES xerogels (with XOx) with and without PU capping layers, along with PU only-modified and bare platinum electrodes during the 100 µL injection of 40 mM XAN at 1200 s and 100 µL injection of CAT enzyme (10 mg/mL) at 2100 s. (**B**) Schematic illustration of critical, complementary role of the PU semi-permeable membrane once H_2_O_2_ is generated within the film.

Constructing the same schemes at platinum electrodes allows for an examination of H_2_O_2_ (Figure 2), the other enzymatic reaction product, though one notoriously difficult to examine, using sweep voltammetry at physiological pH levels [47,48,49,50]. As such, amperometric measurements serve to help probe for mechanistic details in regard to H_2_O_2_. Prior work established that by applying the less oxidating potential of +0.380 V, as opposed to the more commonly reported biosensing potential of +0.65 V [27], one could selectively tune the platinum electrode to be responsive to only H_2_O_2_ oxidation with little to no signal contribution from UA oxidation [37]. Here again, the only source of an electroactive species, in this case predominantly H_2_O_2_, would be derived from the XOx enzymatic reaction within the HMTES xerogel (Figure 1A—Scheme). The results on the GCE electrode suggest that H_2_O_2_ oxidation to be most prevalent at the full system where the PU layer both restricts H_2_O_2_ diffusion away from the electrode and emphasizes the diffusion layer under the PU at the electrode interface. Figure 5A illustrates the typical I-t result observed after an injection of XAN (at ~1200 s) into a hydrodynamic PBS (10 mM; pH 7; stirred) housing a platinum modified with C6 MPC-doped, XOx-embedded HMTES with or without the PU capping layer as well as PU-only modified and bare platinum electrodes for comparison. The steady-state electrochemical responses observed upon the addition of XAN indicate that the most substantial H_2_O_2_ oxidation signal is indeed observed for the PU-capped system—a result consistent with the presented UA result (Figure 4). While the system without the PU-capping layer experiences a significant H_2_O_2_ oxidation response as well, it is smaller in magnitude and not maintained over time, consistent with the loss of H_2_O_2_ away from the electrode and out of the film. Notably, the H_2_O_2_ oxidation at the full system is typically stronger than the bare platinum electrode, resulting in a steadily increasing H_2_O_2_ concentration in the stirred bulk solution. For these experiments, catalase (CAT), an enzyme that efficiently converts H_2_O_2_ to oxygen and water via the reaction below:

(3) $$2H_{2}O_{2}\;\overset{CAT}{\to }\;O_{2}+{2H}_{2}O$$

with the injection of CAT into the solution at ~2100 s (Figure 5A) there are notable responses. Both the PU only and bare platinum electrodes immediately returned to baseline values as H_2_O_2_ was effectively removed from solution while the system without the PU capping layer had a relatively slow response/return to baseline. The most sluggish response to the CAT injection, however, remains the full, PU-capped system. This is likely because the CAT is not as efficient at accessing the H_2_O_2_ being generated within the film, under the PU layer. Additional experiments of this nature and analogous experiments conducted in quiescent solutions are provided in Supporting Information (Figure S7) and show similar results. Briefly, the unstirred experiments show that the XAN injection causes the same effects at each of the electrodes, albeit slower because of the diffusional nature of the experiment. After the injection of XAN, the diffusional responses were delayed and an order-of-magnitude smaller in terms of the measured current, but the same trends persisted with the most significant H_2_O_2_ oxidation occurring most quickly at the PU-capped system. Essentially, these experiments suggest that the signal enhancement at the NP-network is significantly aided by the trapping of the H_2_O_2_ under the PU layer (Figure 5B—Scheme).

**Figure 6:**
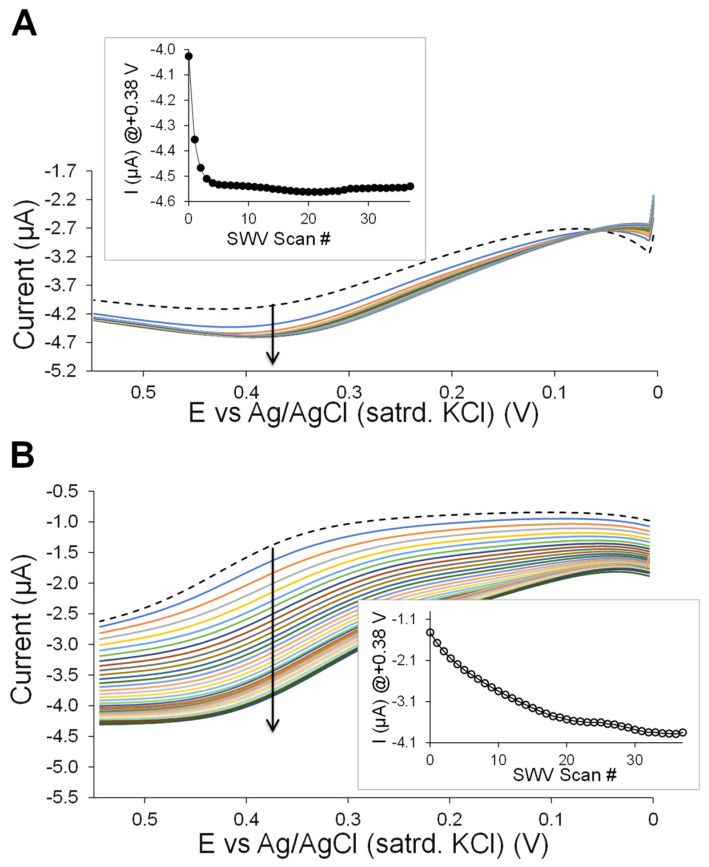
SWV results over time both before (dashed trace) and after (solid traces) the injection of XAN (200 µM) at C6 MPC-doped HMTES (with XOx)-modified platinum electrodes (**A**) with and (**B**) without the PU capping layer with a current measured at +0.380 V over time (inset plots). Note: In terms of time, a typical SWV scan required ~10 s.

One of the more difficult challenges in regard to monitoring traditional enzymatic reactions used in biosensing systems is that H_2_O_2_ electrochemistry is a well-known difficult voltametric measurement [47]. That challenge is further complicated by the fact that H_2_O_2_ generated by the XOx enzyme is at very small concentrations at the modified electrode, where it can also simultaneously diffuse away from the electrode. As such, sweep voltammetry needs to be very sensitive and fast to measure H_2_O_2_ signals. In order to monitor the H_2_O_2_ generated over time at the interface within the film, square wave voltammetry (SWV) was used as it offers several advantages: (1) the ability to sweep potentials fast (seconds vs. minutes with DPV); (2) does not consume redox species at the electrode and; (3) offers lower susceptibility to surface fouling due to rapid positive and negative potential pulses [52]. Figure 6 illustrates representative examples of SWV results, where the platinum electrodes are modified with C6 MPC-doped HMTES xerogels either with (Figure 6A) or without (Figure 6B) the PU capping layer were monitored before and after a XAN injection (100 µL of 40 mM XAN or 200 μM XAN) over time with continuous, sequential SWV sweeps over a potential range, exposing the H_2_O_2_ oxidation current. The current response observed at ~+0.380 V is suspected to be that of H_2_O_2_ at the modified platinum interfaces [44,47,48]. With the same number of scans applied to each system, it is clear the anodic current from H_2_O_2_ oxidation plateaued significantly earlier (~3–4 scans or ~2–3 min, Figure 6A, inset) in the system with the PU layer versus without the PU layer (~30–33 scans or ~12–13 min, Figure 6B, inset). As an additional test of the mechanism and function of the materials, the CAT enzyme (100 µL injection; 1 mg CAT/mL) was again added to the PBS solution and the SWV results monitored with successive repeated scanning. The SWV of the PU-capped system was largely unaffected by the CAT, while the system without PU showed an immediate and sequential signal decrease over time. These SWV overlays are provided in the Supporting Information (Figure S8). The hypothesis is that, because the H_2_O_2_ is generated and efficiently oxidized by the MPC network within the gel and under the PU layer and while the CAT is outside the PU layer, the consumption of H_2_O_2_ is not significant. Indeed, the H_2_O_2_ is fairly persistent even after CAT injection, a result that supports the proposed mechanism of the biosensing system. 

With the critical nature of the PU layer and its critical interplay with the MPC-network established, the only portion of the mechanism left to be observed is the rapid NP to NP electronic communication after H_2_O_2_ oxidation occurs at the MPC network. Prior work has suggested that the electrons are able to rapidly translate through the MPC network to the electrode surface significantly faster than simple molecular diffusion through the film [34,40]. This critical interparticle communication or “electron hopping” is a well-known phenomenon with MPC networks [51] and has been demonstrated with similar MPC-doped xerogel films to those in this study [40]. Experimentally, this interparticle electronic coupling is most easily confirmed if the MPC films demonstrate quantized double layer (QDL) charging peaks during voltammetry sweeps [40,51]. This phenomenon is typically achieved when the films are constructed with MPCs of lower polydispersity [51,53]. As such, the MPCs used in this study were subjected to a well-known fractionation procedure [53] to reduce their polydispersity and were subsequently shown to have QDL charging peaks during DPV scans (Supporting Material, Figure S9A) in a solution (0.1 M TBAP in CH_2_Cl_2_). When the same lower polydispersity material was incorporated into HMTES xerogel and analyzed with DPV, QDL peaks, albeit comparatively difficult to achieve for this system, were visible (Supporting Material, Figure S9B,C), and it could thus be suggested that this part of the mechanism, the efficient interparticle electronic communication, is intact for these films. The fast kinetics of the interparticle “electron hopping” through the MPC film, previously estimated to have a first order rate constant of ~2 × 10^6^ s^−1^, suggests the ability to report redox activity, in this case oxidation of H_2_O_2_, to the electrode at a very fast pace [34,40,51]. 

### 3.2. Carbon Nanotube-Enhanced Xerogel Biosensing Schemes

One of the primary goals of this study was to establish the use of the XAN enzymatic reaction as a general tool for exploring commonly employed biosensors materials that include xerogel scaffolds, outer selective or semi-permeable membranes, different types of electrodes as well as different types and methods of incorporation of NMs for signal enhancement. As such, we used the XAN enzyme system to probe a different system: platinum electrodes modified with single-walled carbon nanotubes with carboxylic acid functionalization (SWCNT-COOH), XOx-embedded xerogels, and PU capping layers (Figure 1A(b)). These layered materials were used in the successful demonstration of a galactose biosensor and share many mechanistic aspects within the film as the previous system with the MPC-network. The major difference is that the SWCNTs are employed directly at the electrode interface rather than directly throughout the three-dimensional xerogel housing the enzyme (Figure 1A(b)). In this situation, the enzyme-generated H_2_O_2_ in the xerogel must still diffuse to the CNT-modified interface to be efficiently oxidized [36].

**Figure 7:**
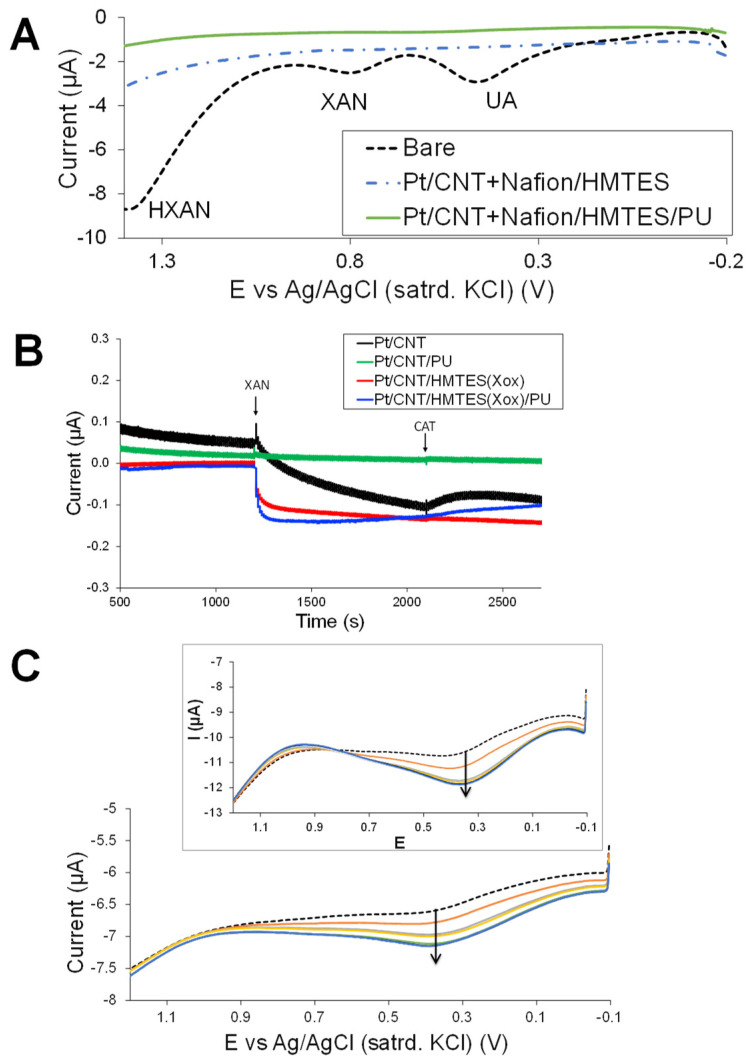
(**A**) Typical DPV results for modified and unmodified (bare) platinum electrodes immersed in a solution of 0.25 mM mixture of HXAN, XAN, and UA (10 mM PBS; pH 7) that illustrate extreme blocking behavior of MPC-doped HMTES xerogel (no XOx) with and without the PU capping layer. (**B**) Amperometric I-t curves of platinum electrodes in PBS (10 mM; pH 7) held at +0.3 V and modified with SWCNT-COOH (Nafion) and HMTES xerogels with and without PU capping layers, along with platinum electrodes modified with SWCNT-COOH (Nafion) with and without PU capping layers (i.e., no HMTES xerogels) during 100 µL injection of 40 mM XAN at 1200 s and 100 µL injection of CAT enzyme (10 mg/mL) at 2100 s. (**C**) SWV results over time before (dashed trace) and after (solid traces) injection of XAN (200 µM) at platinum electrodes modified with SWCNT-COOHs (Nafion) and HMTES xerogels (with XOx) with and without (inset) the PU capping layer. Note: In terms of time, a typical SWV scan was ~10 s.

For this type of scheme, the XAN enzymatic reaction, producing both UA and H_2_O_2_ within the HMTES xerogel beneath the PU layer, was utilized, as previously shown, in order to study this scheme’s mechanism (Figure 1B—Scheme). First, however, the DPV analysis (Figure 7A) of the SWCNT(COOH)-modified platinum electrodes with and without the PU capping layer again illustrate that the PU membrane creates two distinctive types of diffusional layers. The redox activity of diffusional species in bulk solution outside the PU membrane, a mixture of UA, XAN, and HXAN, is exclusively blocked from electrode access compared to a bare platinum electrode. A similar phenomenon was also observed using CV and SWV (Supporting Information, Figure S10). 

Given that different types of modified electrodes can electro-catalyze different reactions at different potentials, the optimal hold potential was again determined through a series of standard injections aimed at finding a potential that would maximize the oxidation of H_2_O_2_ and minimize the signal from UA at the CNT-modified platinum electrodes. Amperometric I-t curves generated with the modified platinum electrode held at +0.30 V were determined as optimal for monitoring the H_2_O_2_ signal (Supporting Information, Figure S11). As previously conducted on the MPC system (Figure 5), Figure 7B provides an example of the amperometric I-t analysis of the CNT-based systems, held at the optimized +0.3 V during injections of XAN and CAT. Here, in this system, we again see the critical role of the outer semi-permeable membrane (PU) interplay with the NM-enhancement effect. The I-t response to injected XAN is most pronounced with the PU-capped HMTES (XOx) system at SWCNT-modified platinum, likely because the H_2_O_2_ generated is partially trapped under the PU layer. Without the PU layer, the response at XAN injection, i.e., H_2_O_2_ oxidation, is both smaller in magnitude and slower. As expected, a system lacking XOx, a SWCNT-modified platinum with a PU capping layer, is unresponsive to the injections, sufficiently blocking electroactive species in the solution and generating no H_2_O_2_ under the PU layer. The SWCNT-Pt electrode without PU slowly accumulates the gathering H_2_O_2_ in solution as it leaks from the other systems, as evidenced by the slow increase in anodic current, followed by an abrupt decrease in current with the addition of CAT and the subsequent slower increase in anodic current, a classic example of the competition between the oxidation of leaking H_2_O_2_ and the CAT consumption of H_2_O_2_ [36].

As previously demonstrated in this study with the NP-doped system, the SWV analysis of H_2_O_2_ oxidation with the CNT-based systems was again applied and resulted in a similar, albeit not as pronounced, effect. Repeated SWV scans before and after injecting the solution with XAN at these CNT-based systems with and without the PU layer are shown in Figure 7C. In these systems, we again observe a fast build-up of H_2_O_2_ under the PU layer that is quickly oxidized at the electrode (Figure 7C) and a slower build-up when that PU layer is absent (Figure 7C, inset), respectively. We can suggest two reasons why the effect, while still evident, is markedly less pronounced and slower (i.e., ~14 scans; ~12 min). First, the two systems are run together in the same solution (i.e., multi-channel potentiostat with a common RE), making it likely that there is significant H_2_O_2_ leakage to the bulk solution from the uncapped system. Accentuating this phenomenon, is the fact that these CNT-based systems still require significantly more H_2_O_2_ diffusion to take place. Once generated by the enzymatic reaction, H_2_O_2_ must still diffuse through the xerogel to reach the SWCNT/platinum interface and may also diffuse away from the electrode as well. As such, the additional time required and the corresponding diffusional leakage from both systems may be more similar and result in a less substantial signal enhancement effect attributable to the PU outer layer. It is also notable that the system without the PU passes higher current by allowing considerable access to the xerogel. 

As with the MPC-network system (Figure 6), SWV after the addition of CAT to these SWCNT systems with and without the PU layer was of interest. In this case, prior to adding CAT, standardized H_2_O_2_ was injected (100 µL injection of 30% H_2_O_2_) into the system to see what effect it had on the voltammetry of enzyme-generated H_2_O_2_ oxidation. In both systems, with and without the PU capping layer, the injection of standard H_2_O_2_ (non-enzyme-generated) had little effect on the voltammetry on the timescale of the SWV. That is, both systems are more sensitive to the H_2_O_2_ being generated within the films and oxidized at the SWCNT interface rather than diffusion from bulk solution (i.e., the PU and/or the HMTES xerogel are blocking toward solution species as expected). With the addition of CAT to the systems, we see a more dramatic effect of H_2_O_2_ consumption that might be attributed to the more porous xerogels in this system (un-doped with the MPCs)—see Supporting Information, Figure S12.

## 4. Conclusions

The collective results of this study suggest that the enzymatic reaction of XAN with XOx, when applied to both these NM-augmented schemes and strategies, shows that signal enhancement is achieved when the incorporated NMs work in concert with the semi-permeable membrane to redefine the diffusion layer of a modified electrode. In doing so, the materials work together to condense the electrochemical interface through improved electronic communication and coupling, or as the result of improving the flux of electroactive species toward the working electrode (transducer). Because the enzymatic reactions of this nature involve electroactive products and reactants, it provides an opportunity to assess the electrochemistry, not only traditionally, as a diffusing species from bulk solution, but also within the layers of a modified electrode where the reaction generates multiple species sensitive to both applied potential and interfacial materials. The commonality of both the NMs and membranes used in this study to numerous biosensors suggests that the enzymatic reactions of this nature could find more widespread application in understanding the functionality of materials toward effective biosensing. 

## Data Availability

The data presented in this study are available on request from the corresponding author.

## Supplementary Materials

The following supporting information can be downloaded at: https://www.mdpi.com/article/10.3390/bios13080798/s1, Figure S1: I-t and calibration curves for signal enhancement at CNT-modified xerogel electrodes; Experimental Details: Typical electrochemical parameters for sweep voltammetry techniques used in the study (DPV, SWV); Scheme S1: Schematic of xanthine metabolism enzymatic reaction and its electroactive products; Figure S2: Sweep voltammetry (CV, DPV, SWV) of a mixture of XAN, HXAN, and UA at a GCE interface; Figure S3: Amperometric I-t analysis analogous to Figure 2 in reverse order of injections at Pt vs. GCE electrodes; Figure S4: CV voltammetry of H_2_O_2_ at a bare platinum electrode; Figures S5 and S6: DPV scans over time shown in Figure 4 but also including scans taken 1 h (and long) after XAN injection (GCEs modified with C6-MPC doped HMTES xerogels (with XOx) with and without PU capping layer; Figure S7: Amperometric I-t analysis during injection of XAN and CAT in an unstirred solution (Figure 5A); Figure S8: SWV over time for C6-MPC doped HMTES system with and without PU (Figure 6) including scans taken after the addition of CAT; Figure S9: DPV scans of C6 MPC in solution and embedded in HMTES xerogels; Figure S10: Sweep voltammetry (CV, SWV) of a mixture of XAN, HXAN, and UA at a Pt/COOH-SWCNT (Nafion)/HMTES system with and without PU outer layer; Figure S11: Amperometric I-t analysis to determine optimal potential to apply to Pt/COOH-SWCNT (Nafion) systems to gain selectivity and sensitivity towards H_2_O_2_ oxidation; Figure S12: SWV over time for Pt/SWCNT-COOH (Nafion)/HMTES (XOx) system with and without PU (Figure 7) including scans taken after the addition of either H_2_O_2_ or CAT.

## Abbreviations

CAT—catalase enzyme; CE—platinum auxiliary electrode; CNT—carbon based nanotubes; CV—cyclic voltammetry; DPV—differential pulse voltammetry; GCE—glassy carbon electrode; HMTES—hydroxymethyl triethoxy silane; HPU—hydrothane polyurethane; HXAN—hypoxanthine; LbL—layer by layer; C6 MPC—hexanethiolate monolayer protected clusters; NM—nanomaterials; NP—nanoparticles; PBS—potassium phosphate buffer; Pt—platinum; PU—polyurethane; RE—reference electrode; SWCNT (COOH)—single walled carbon nanotubes with carboxylic acid functionalization; SWV—square wave voltammetry; TBAP—tetrabutylammonium perchlorate; THF—tetrahydrofuran; UA—uric acid; UP H_2_O—ultra pure water; XAN—xanthine; XOx—xanthine oxidase.
