# Supplementary material for: Mechanistic Elucidation of Nanomaterial-Enhanced First-Generation Biosensors Using Probe Voltammetry of an Enzymatic Reaction
Source: Biosensors (Basel). 2023 Aug 9;13(8):798. doi: 10.3390/bios13080798 (PMC10452687; doi:10.3390/bios13080798)
Supplement: Supplementary file 1 [file biosensors-13-00798-s001.zip › biosensors-2535685-supplementary.pptx]

## Slide 1
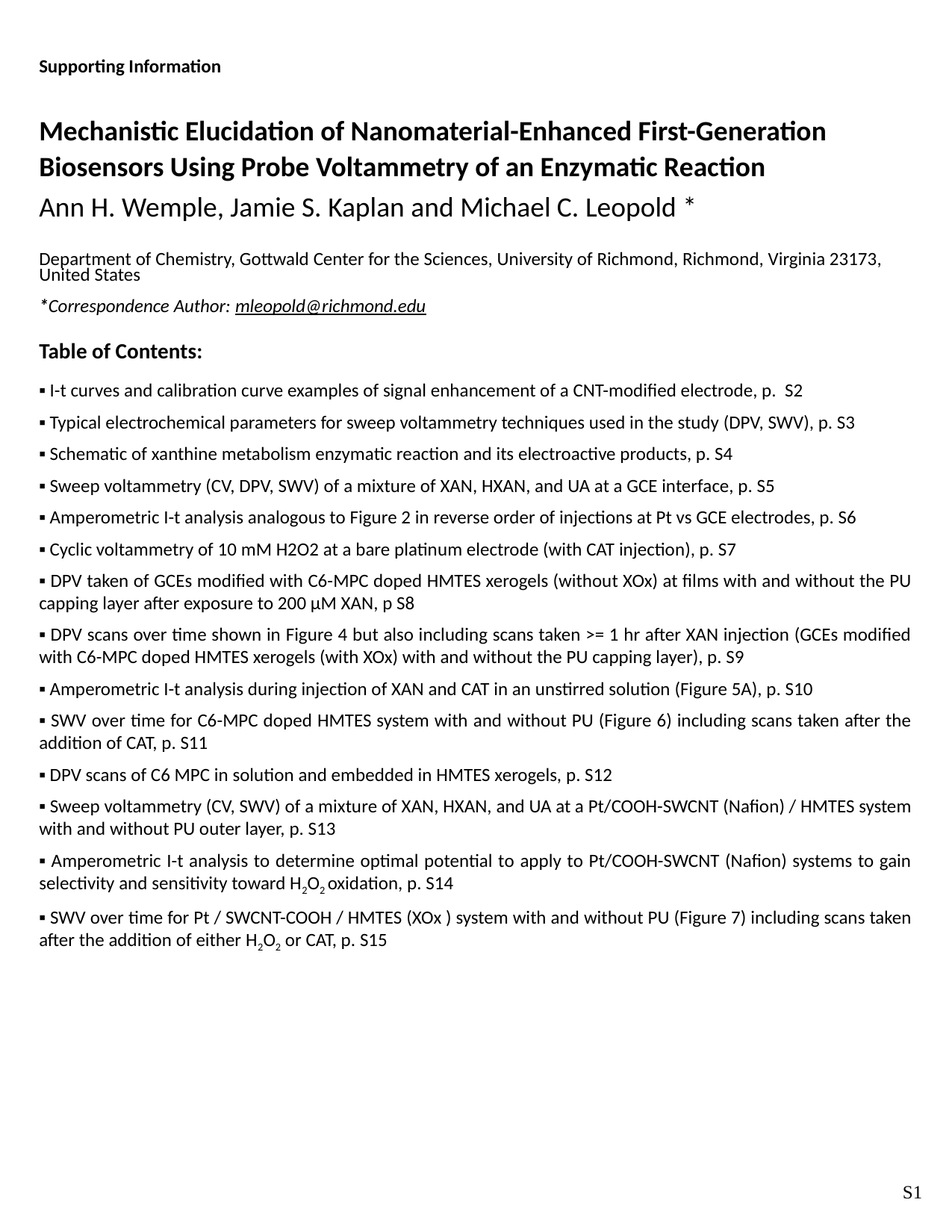

Supporting Information
Mechanistic Elucidation of Nanomaterial-Enhanced First-Generation Biosensors Using Probe Voltammetry of an Enzymatic Reaction
Ann H. Wemple, Jamie S. Kaplan and Michael C. Leopold *
Department of Chemistry, Gottwald Center for the Sciences, University of Richmond, Richmond, Virginia 23173, United States
*Correspondence Author: mleopold@richmond.edu
Table of Contents:
▪ I-t curves and calibration curve examples of signal enhancement of a CNT-modified electrode, p. S2
▪ Typical electrochemical parameters for sweep voltammetry techniques used in the study (DPV, SWV), p. S3
▪ Schematic of xanthine metabolism enzymatic reaction and its electroactive products, p. S4
▪ Sweep voltammetry (CV, DPV, SWV) of a mixture of XAN, HXAN, and UA at a GCE interface, p. S5
▪ Amperometric I-t analysis analogous to Figure 2 in reverse order of injections at Pt vs GCE electrodes, p. S6
▪ Cyclic voltammetry of 10 mM H2O2 at a bare platinum electrode (with CAT injection), p. S7
▪ DPV taken of GCEs modified with C6-MPC doped HMTES xerogels (without XOx) at films with and without the PU capping layer after exposure to 200 µM XAN, p S8
▪ DPV scans over time shown in Figure 4 but also including scans taken >= 1 hr after XAN injection (GCEs modified with C6-MPC doped HMTES xerogels (with XOx) with and without the PU capping layer), p. S9
▪ Amperometric I-t analysis during injection of XAN and CAT in an unstirred solution (Figure 5A), p. S10
▪ SWV over time for C6-MPC doped HMTES system with and without PU (Figure 6) including scans taken after the addition of CAT, p. S11
▪ DPV scans of C6 MPC in solution and embedded in HMTES xerogels, p. S12
▪ Sweep voltammetry (CV, SWV) of a mixture of XAN, HXAN, and UA at a Pt/COOH-SWCNT (Nafion) / HMTES system with and without PU outer layer, p. S13
▪ Amperometric I-t analysis to determine optimal potential to apply to Pt/COOH-SWCNT (Nafion) systems to gain selectivity and sensitivity toward H2O2 oxidation, p. S14
▪ SWV over time for Pt / SWCNT-COOH / HMTES (XOx ) system with and without PU (Figure 7) including scans taken after the addition of either H2O2 or CAT, p. S15
S1

## Slide 2
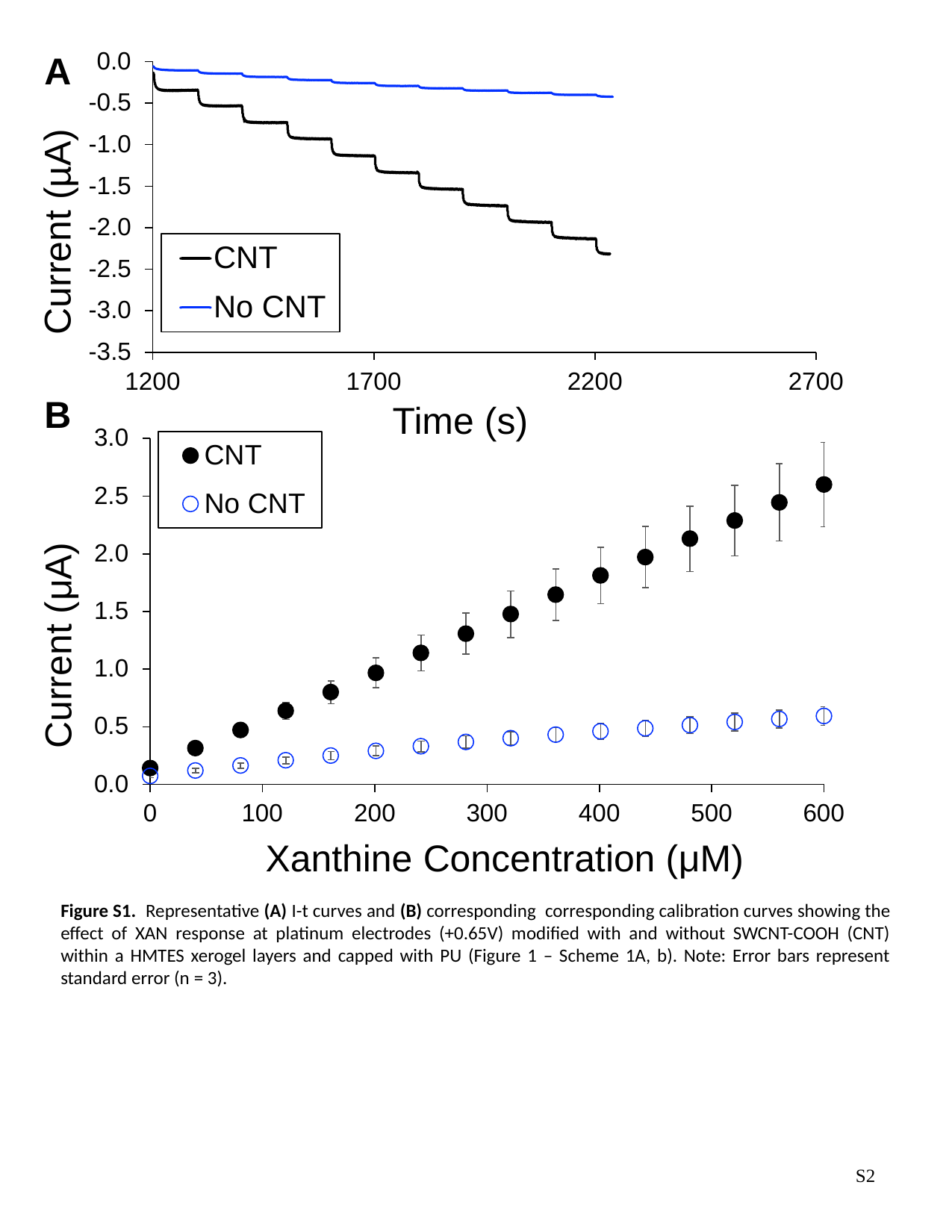

Figure S1. Representative (A) I-t curves and (B) corresponding corresponding calibration curves showing the effect of XAN response at platinum electrodes (+0.65V) modified with and without SWCNT-COOH (CNT) within a HMTES xerogel layers and capped with PU (Figure 1 – Scheme 1A, b). Note: Error bars represent standard error (n = 3).
S2

## Slide 3
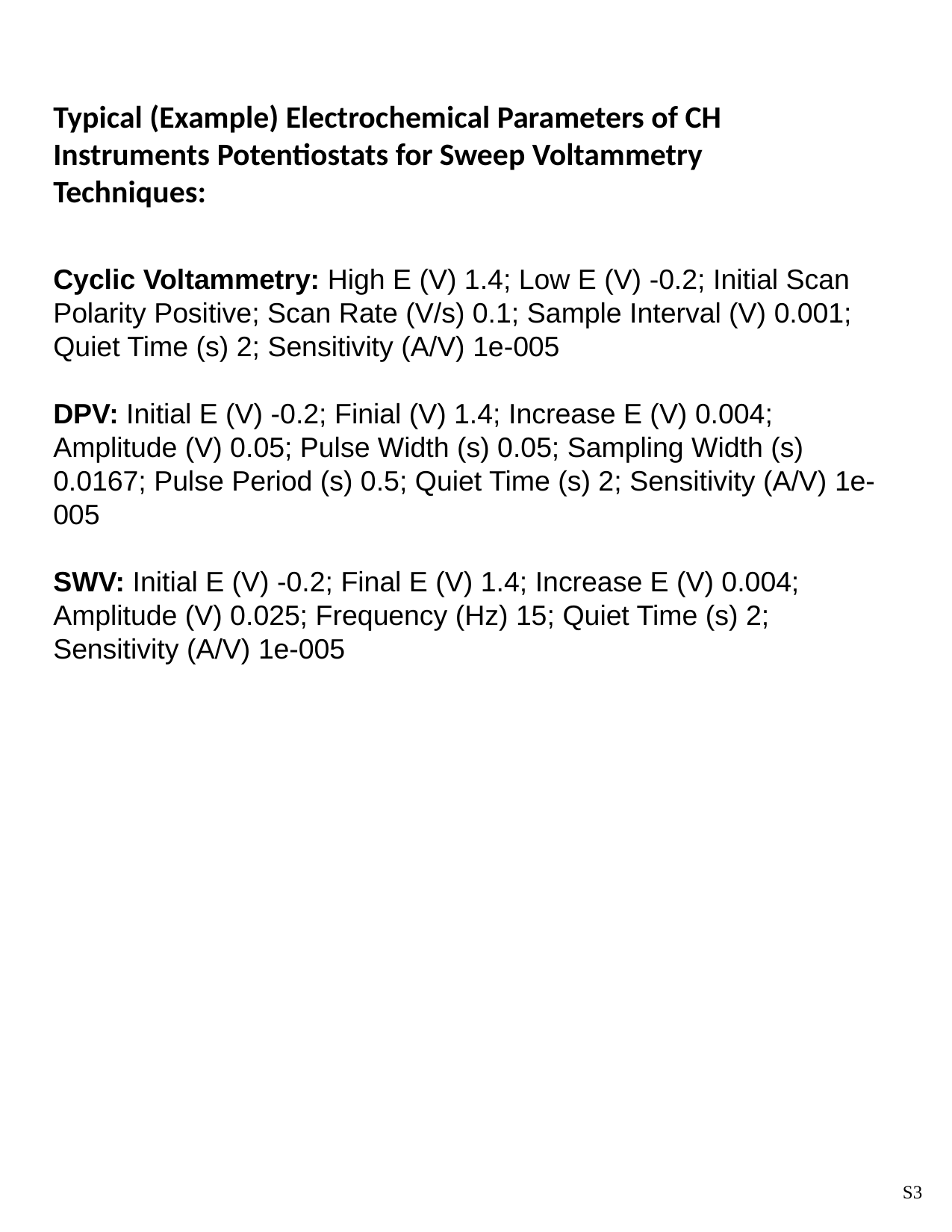

Typical (Example) Electrochemical Parameters of CH Instruments Potentiostats for Sweep Voltammetry Techniques:
Cyclic Voltammetry: High E (V) 1.4; Low E (V) -0.2; Initial Scan Polarity Positive; Scan Rate (V/s) 0.1; Sample Interval (V) 0.001; Quiet Time (s) 2; Sensitivity (A/V) 1e-005
DPV: Initial E (V) -0.2; Finial (V) 1.4; Increase E (V) 0.004; Amplitude (V) 0.05; Pulse Width (s) 0.05; Sampling Width (s) 0.0167; Pulse Period (s) 0.5; Quiet Time (s) 2; Sensitivity (A/V) 1e-005
SWV: Initial E (V) -0.2; Final E (V) 1.4; Increase E (V) 0.004; Amplitude (V) 0.025; Frequency (Hz) 15; Quiet Time (s) 2; Sensitivity (A/V) 1e-005
S3

## Slide 4
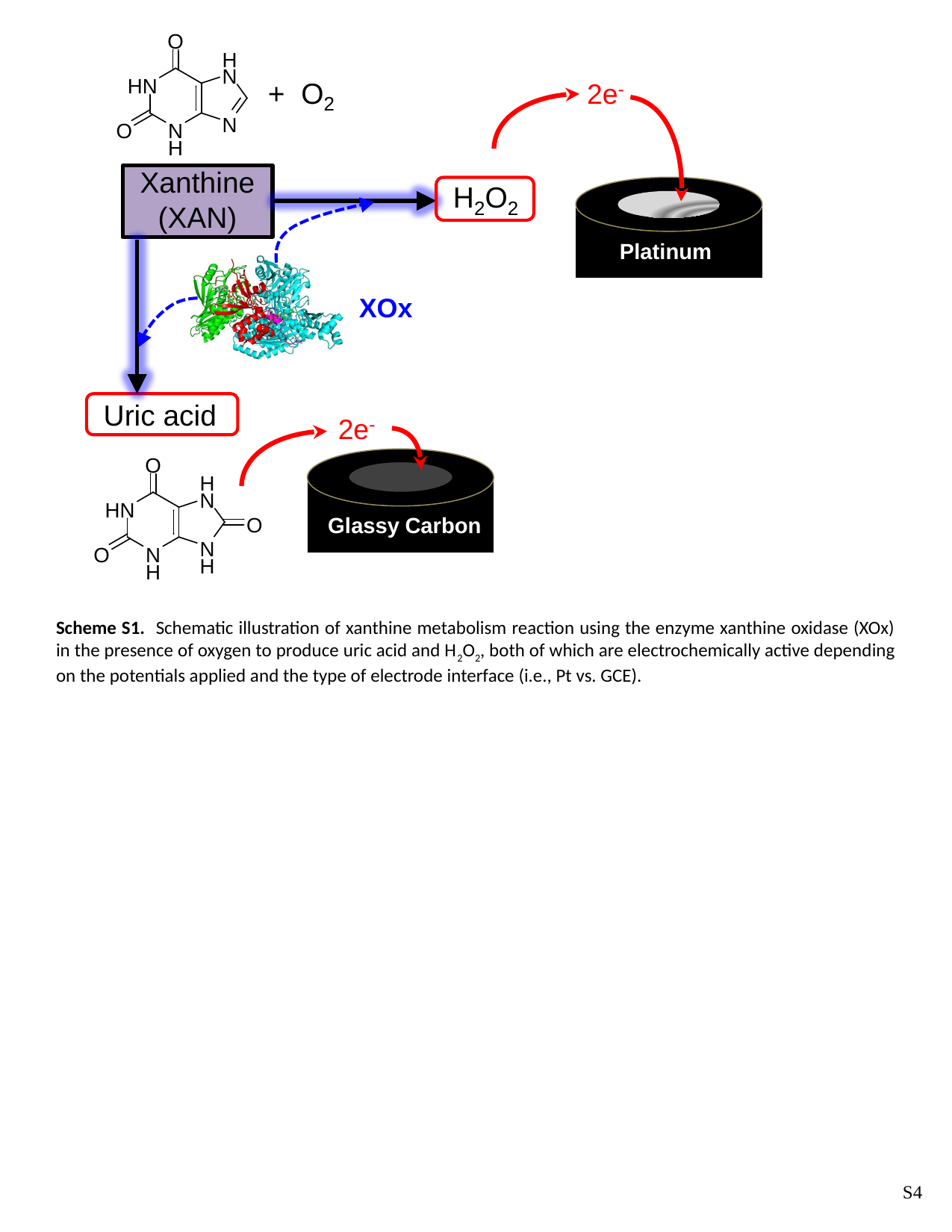

Scheme S1. Schematic illustration of xanthine metabolism reaction using the enzyme xanthine oxidase (XOx) in the presence of oxygen to produce uric acid and H2O2, both of which are electrochemically active depending on the potentials applied and the type of electrode interface (i.e., Pt vs. GCE).
S4

## Slide 5
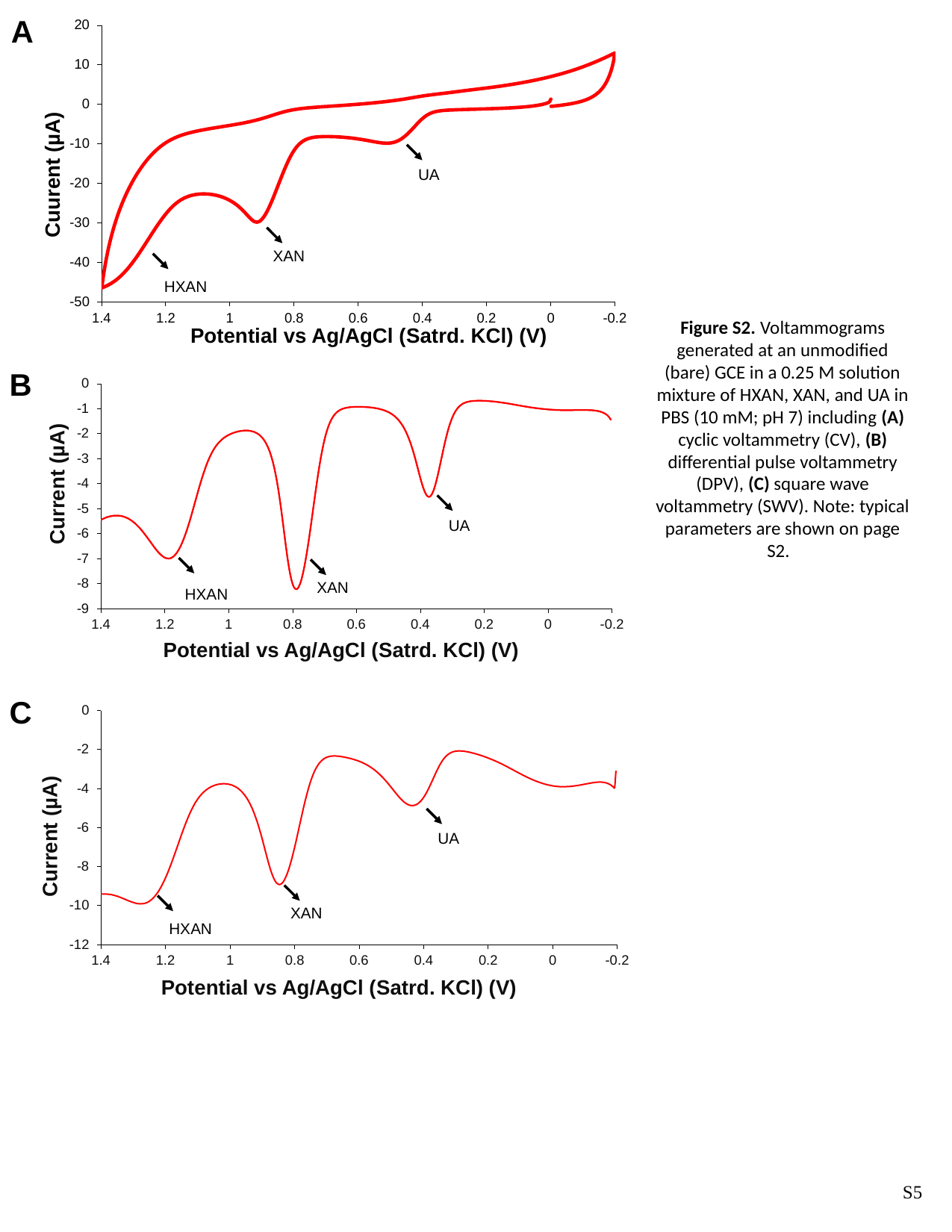

A
Figure S2. Voltammograms generated at an unmodified (bare) GCE in a 0.25 M solution mixture of HXAN, XAN, and UA in PBS (10 mM; pH 7) including (A) cyclic voltammetry (CV), (B) differential pulse voltammetry (DPV), (C) square wave voltammetry (SWV). Note: typical parameters are shown on page S2.
B
C
S5

## Slide 6
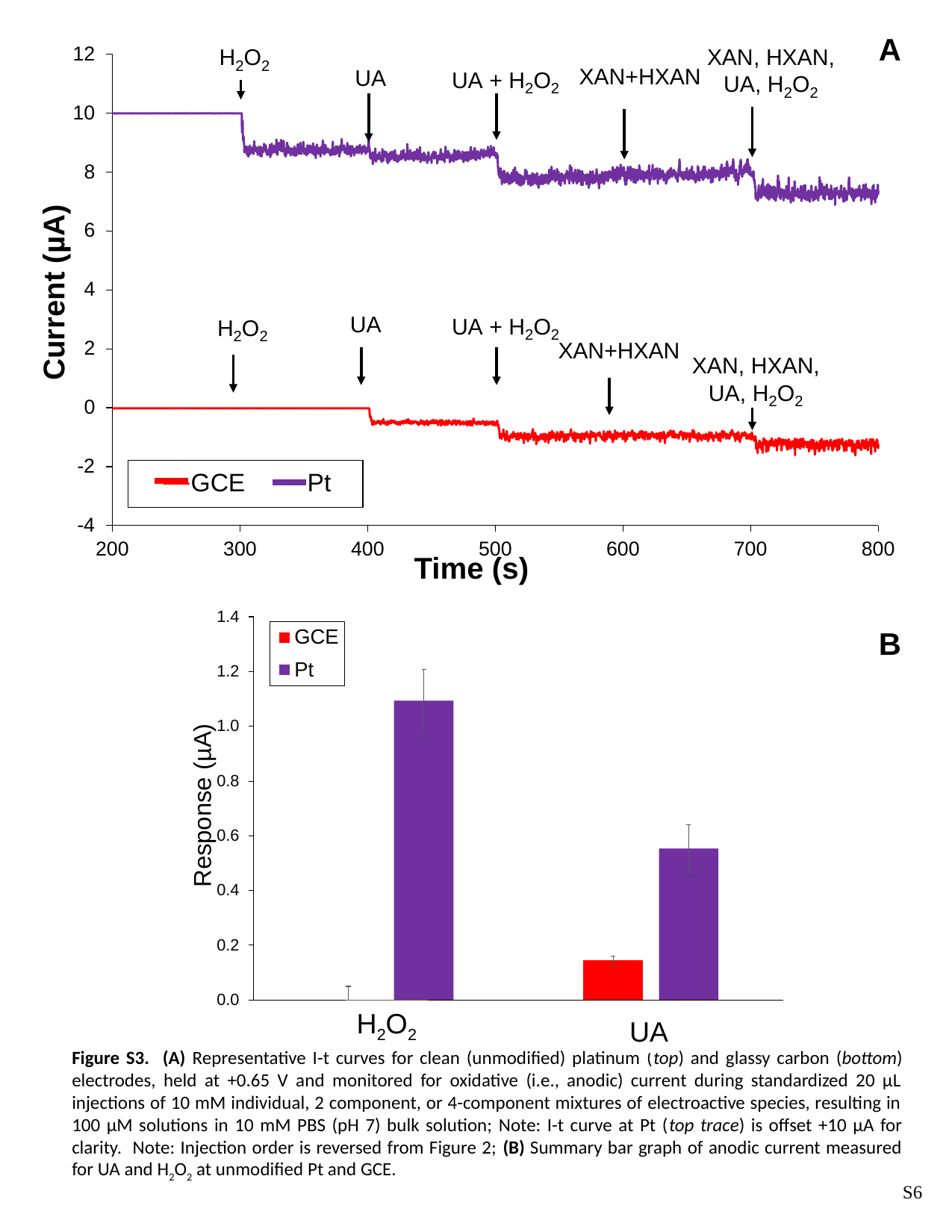

A
B
H2O2
UA
Figure S3. (A) Representative I-t curves for clean (unmodified) platinum (top) and glassy carbon (bottom) electrodes, held at +0.65 V and monitored for oxidative (i.e., anodic) current during standardized 20 µL injections of 10 mM individual, 2 component, or 4-component mixtures of electroactive species, resulting in 100 µM solutions in 10 mM PBS (pH 7) bulk solution; Note: I-t curve at Pt (top trace) is offset +10 µA for clarity. Note: Injection order is reversed from Figure 2; (B) Summary bar graph of anodic current measured for UA and H2O2 at unmodified Pt and GCE.
S6

## Slide 7
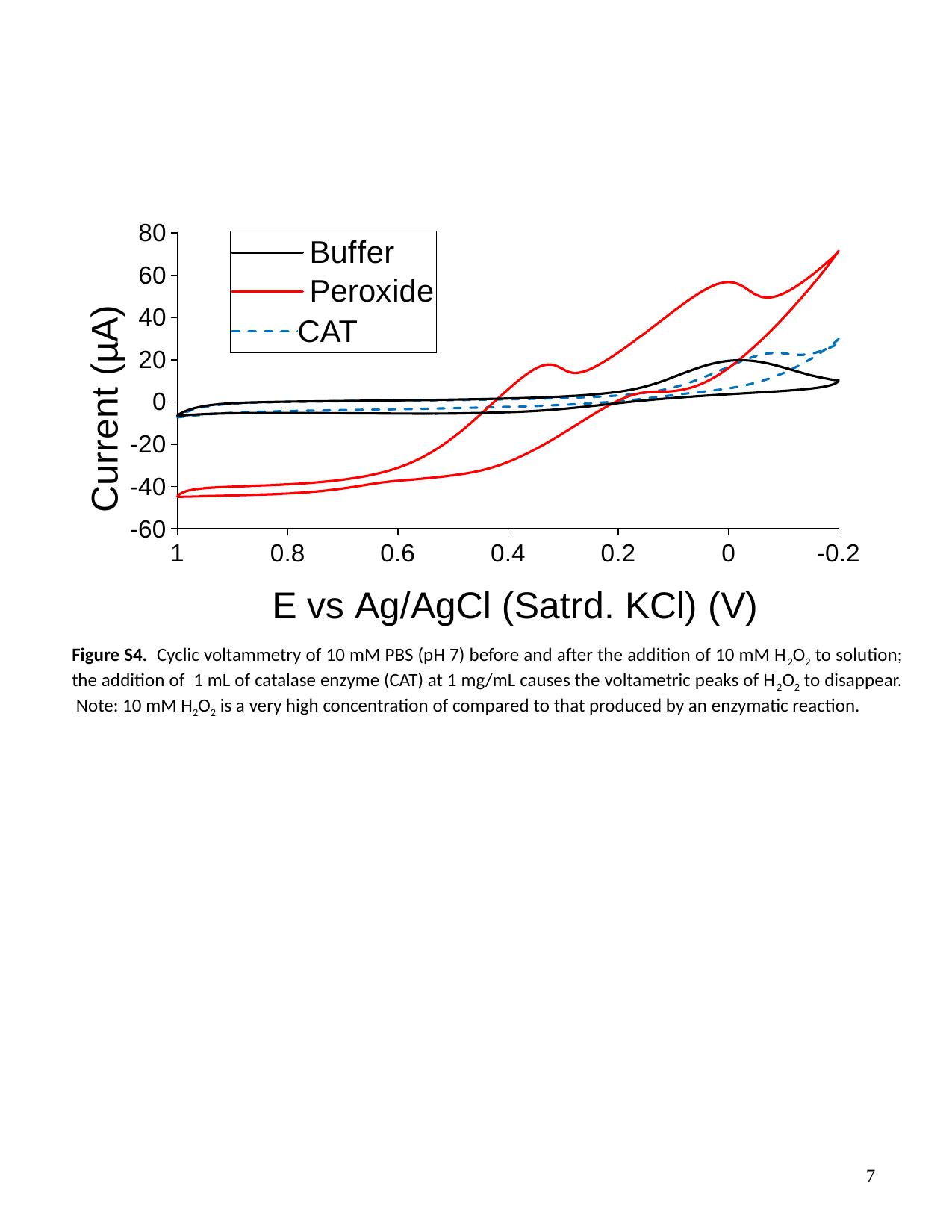

### Chart
| Category | Buffer | Peroxide | Cat |
|---|---|---|---|CAT
Figure S4. Cyclic voltammetry of 10 mM PBS (pH 7) before and after the addition of 10 mM H2O2 to solution; the addition of 1 mL of catalase enzyme (CAT) at 1 mg/mL causes the voltametric peaks of H2O2 to disappear. Note: 10 mM H2O2 is a very high concentration of compared to that produced by an enzymatic reaction.
7

## Slide 8
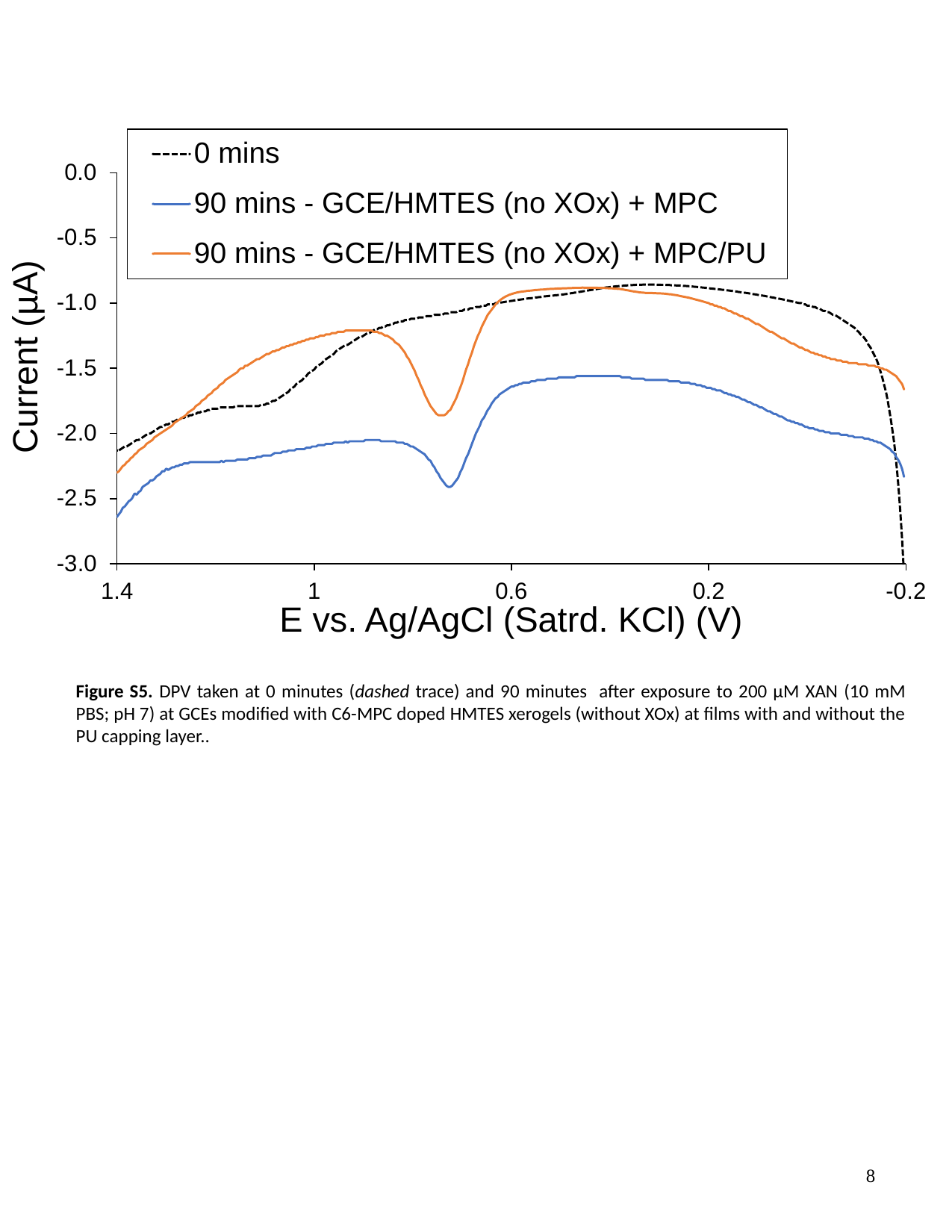

Figure S5. DPV taken at 0 minutes (dashed trace) and 90 minutes after exposure to 200 µM XAN (10 mM PBS; pH 7) at GCEs modified with C6-MPC doped HMTES xerogels (without XOx) at films with and without the PU capping layer..
8

## Slide 9
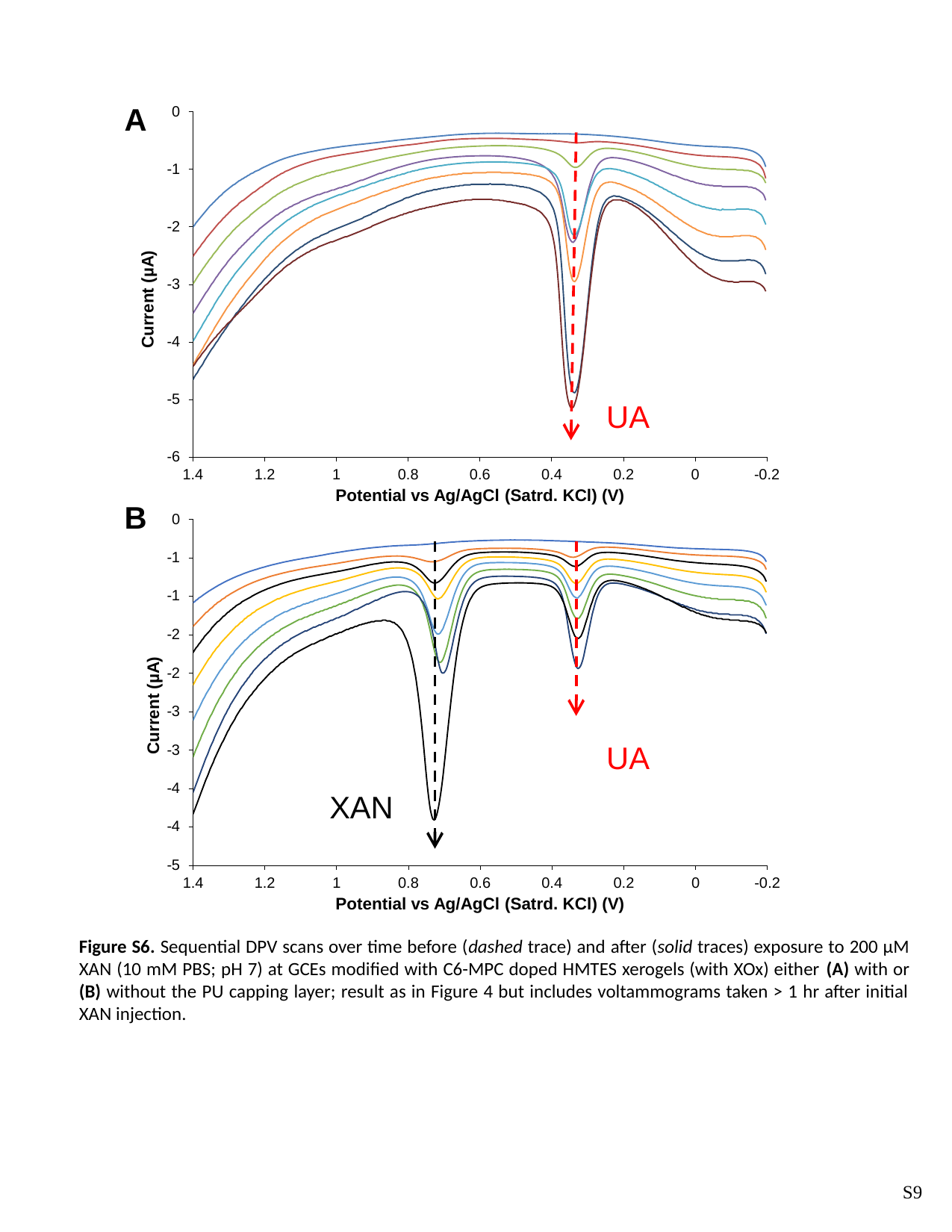

A
UA
B
UA
XAN
Figure S6. Sequential DPV scans over time before (dashed trace) and after (solid traces) exposure to 200 µM XAN (10 mM PBS; pH 7) at GCEs modified with C6-MPC doped HMTES xerogels (with XOx) either (A) with or (B) without the PU capping layer; result as in Figure 4 but includes voltammograms taken > 1 hr after initial XAN injection.
S9

## Slide 10
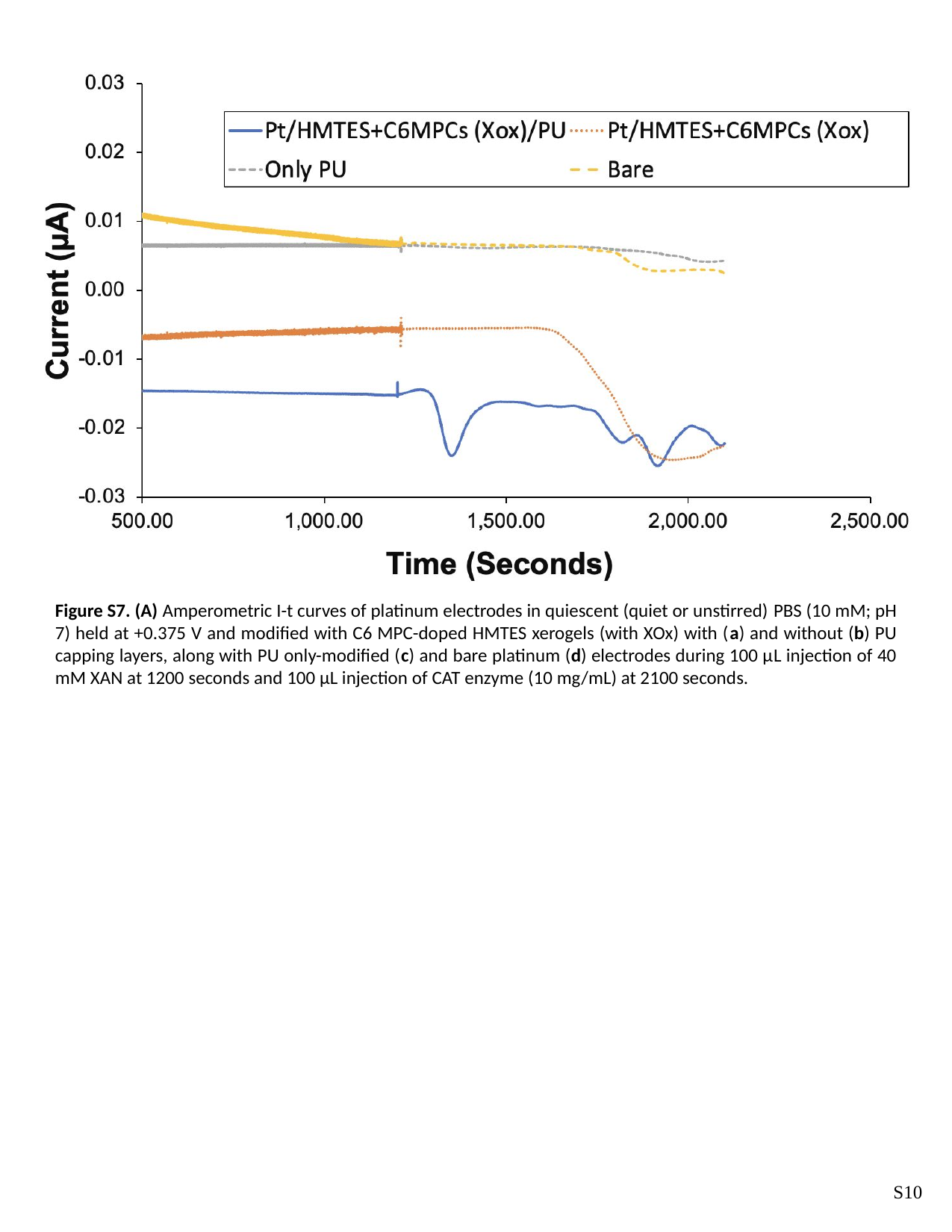

Figure S7. (A) Amperometric I-t curves of platinum electrodes in quiescent (quiet or unstirred) PBS (10 mM; pH 7) held at +0.375 V and modified with C6 MPC-doped HMTES xerogels (with XOx) with (a) and without (b) PU capping layers, along with PU only-modified (c) and bare platinum (d) electrodes during 100 µL injection of 40 mM XAN at 1200 seconds and 100 µL injection of CAT enzyme (10 mg/mL) at 2100 seconds.
S10

## Slide 11
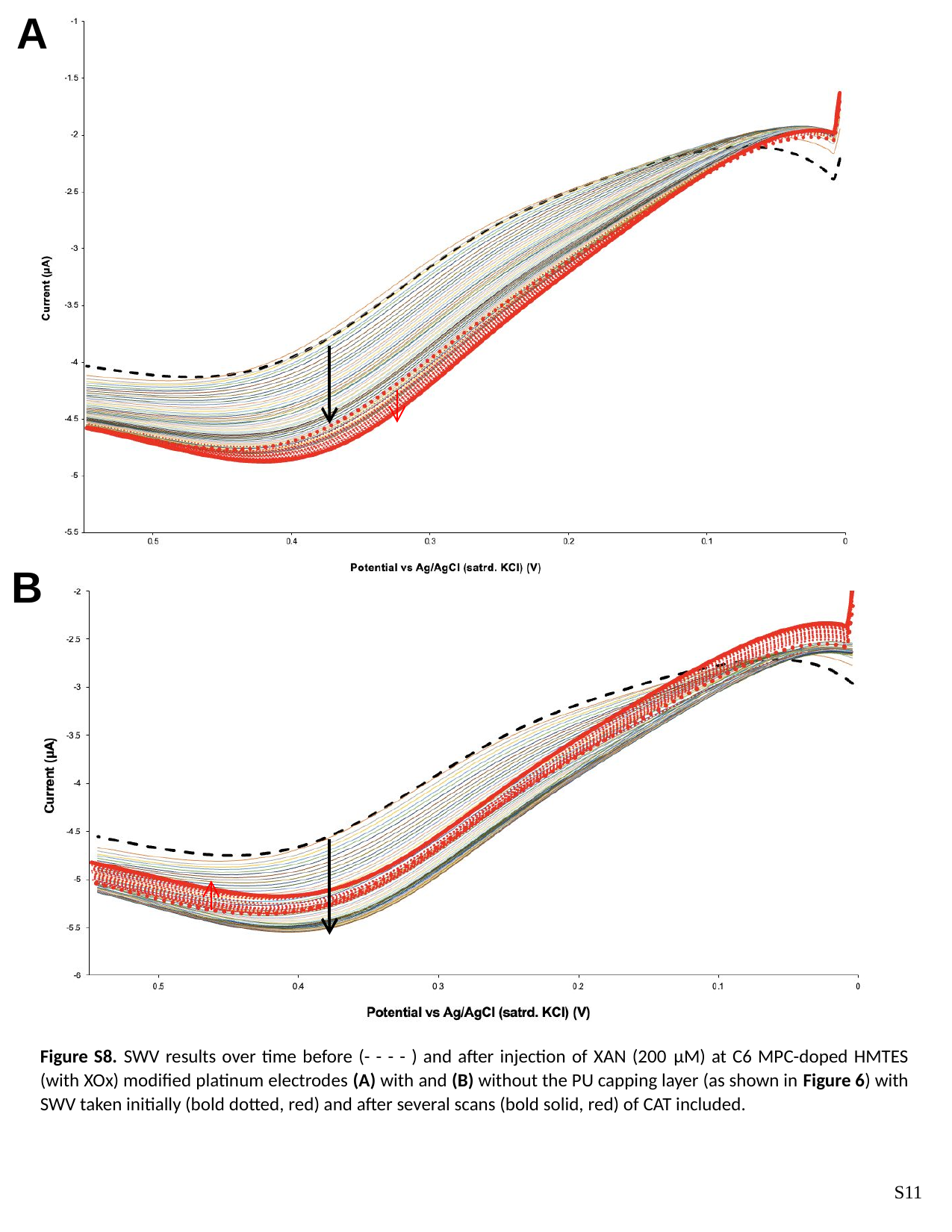

A
B
Figure S8. SWV results over time before (- - - - ) and after injection of XAN (200 µM) at C6 MPC-doped HMTES (with XOx) modified platinum electrodes (A) with and (B) without the PU capping layer (as shown in Figure 6) with SWV taken initially (bold dotted, red) and after several scans (bold solid, red) of CAT included.
S11

## Slide 12
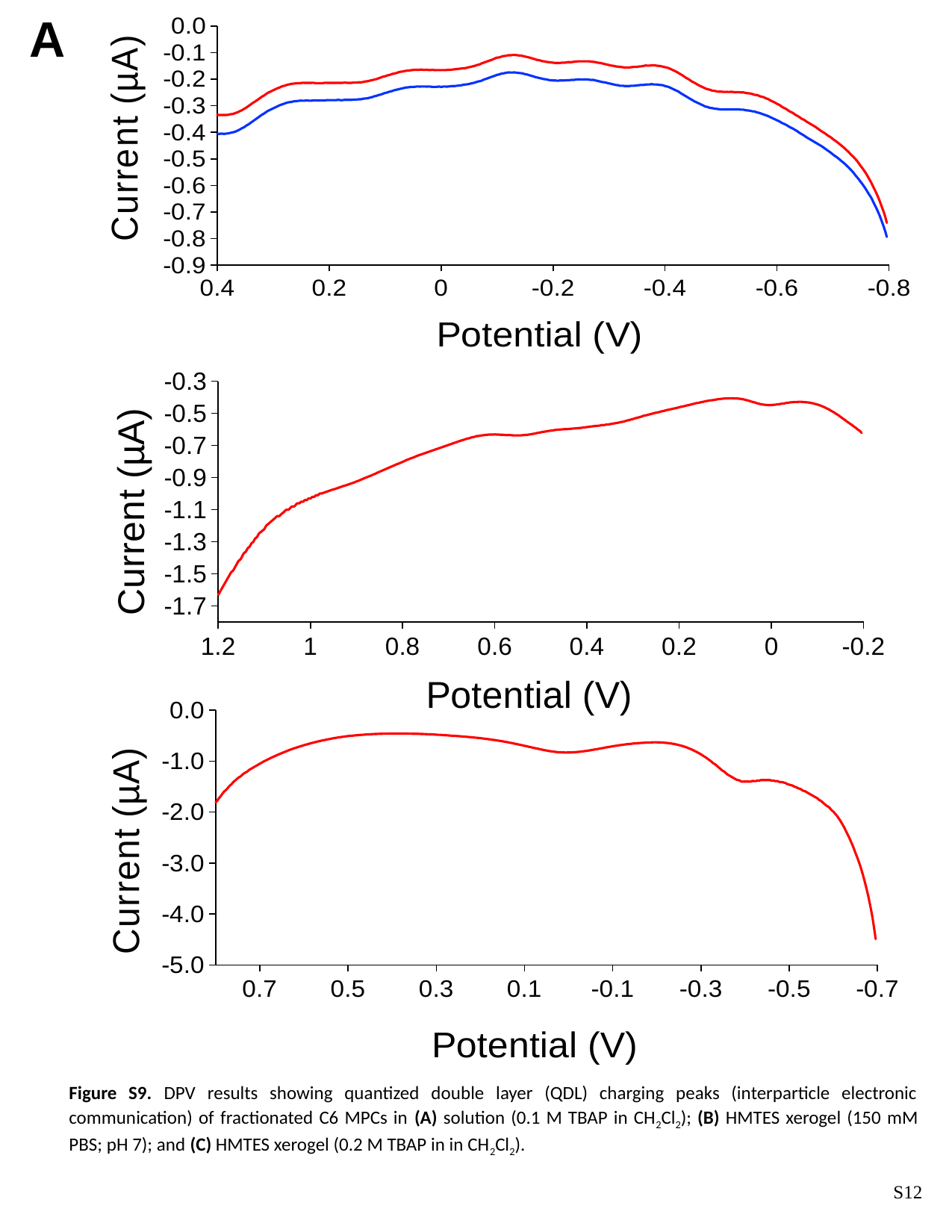

A
### Chart
| Category | Current | |
|---|---|---|
### Chart
| Category | |
|---|---|
### Chart
| Category | Current |
|---|---|Figure S9. DPV results showing quantized double layer (QDL) charging peaks (interparticle electronic communication) of fractionated C6 MPCs in (A) solution (0.1 M TBAP in CH2Cl2); (B) HMTES xerogel (150 mM PBS; pH 7); and (C) HMTES xerogel (0.2 M TBAP in in CH2Cl2).
S12

## Slide 13
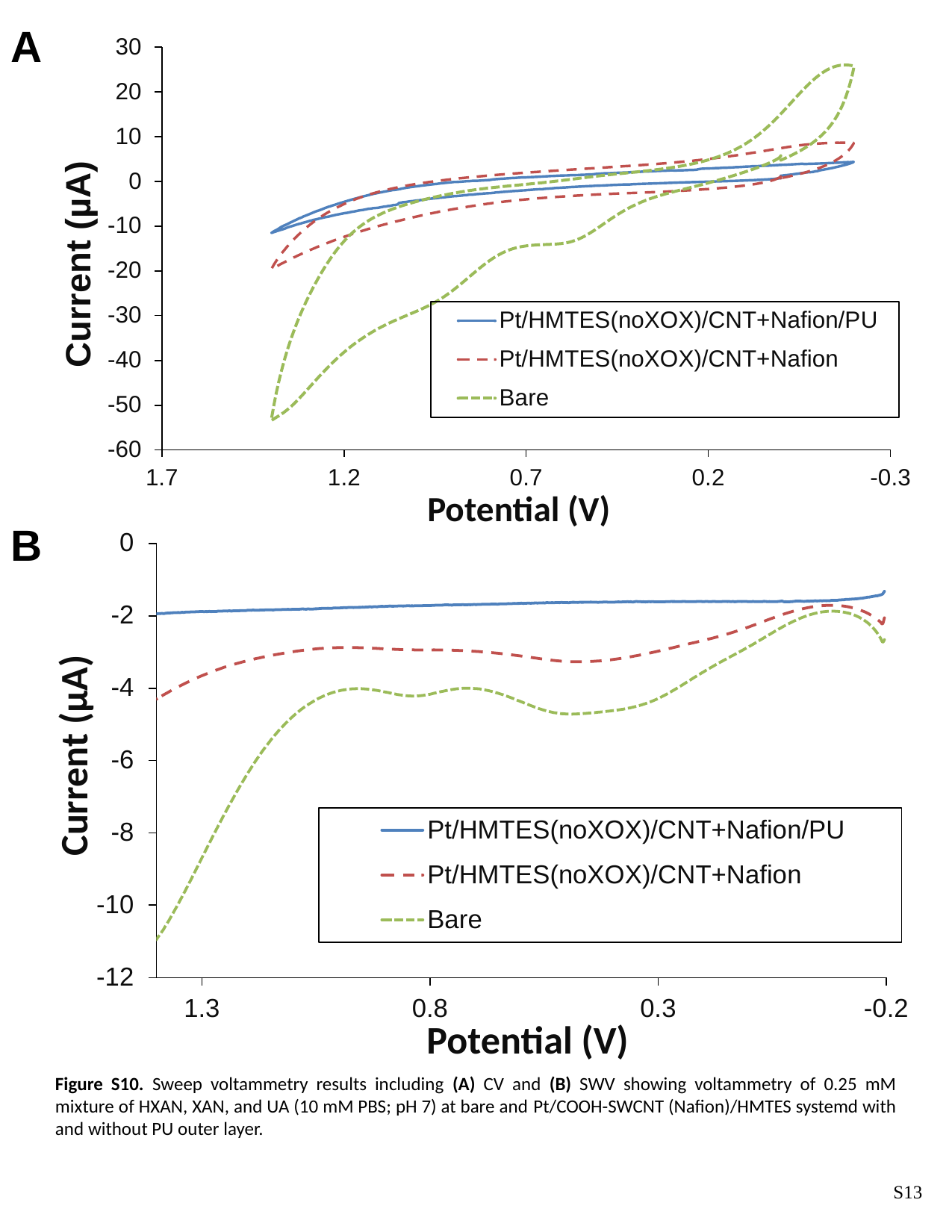

A
B
Figure S10. Sweep voltammetry results including (A) CV and (B) SWV showing voltammetry of 0.25 mM mixture of HXAN, XAN, and UA (10 mM PBS; pH 7) at bare and Pt/COOH-SWCNT (Nafion)/HMTES systemd with and without PU outer layer.
S13

## Slide 14
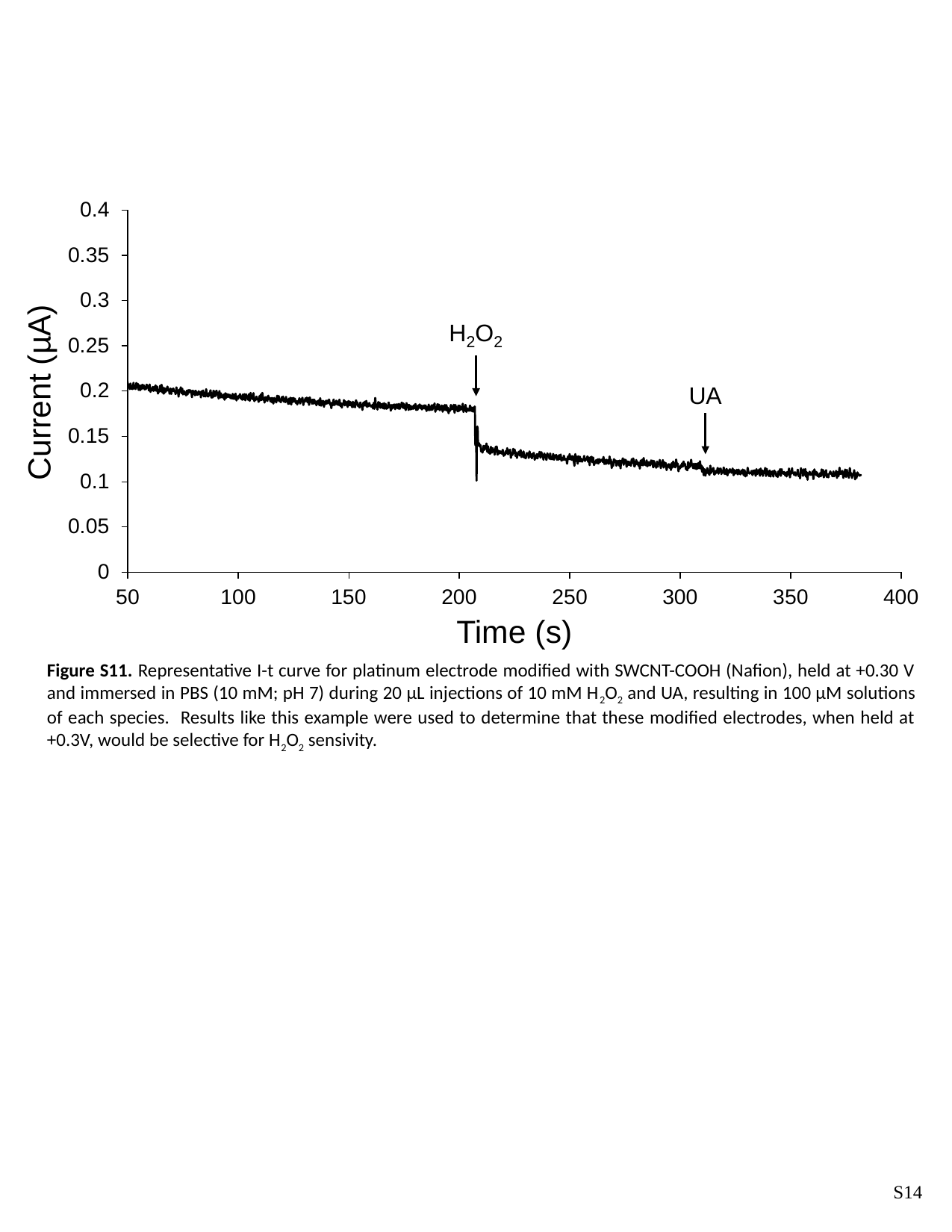

Figure S11. Representative I-t curve for platinum electrode modified with SWCNT-COOH (Nafion), held at +0.30 V and immersed in PBS (10 mM; pH 7) during 20 µL injections of 10 mM H2O2 and UA, resulting in 100 µM solutions of each species. Results like this example were used to determine that these modified electrodes, when held at +0.3V, would be selective for H2O2 sensivity.
S14

## Slide 15
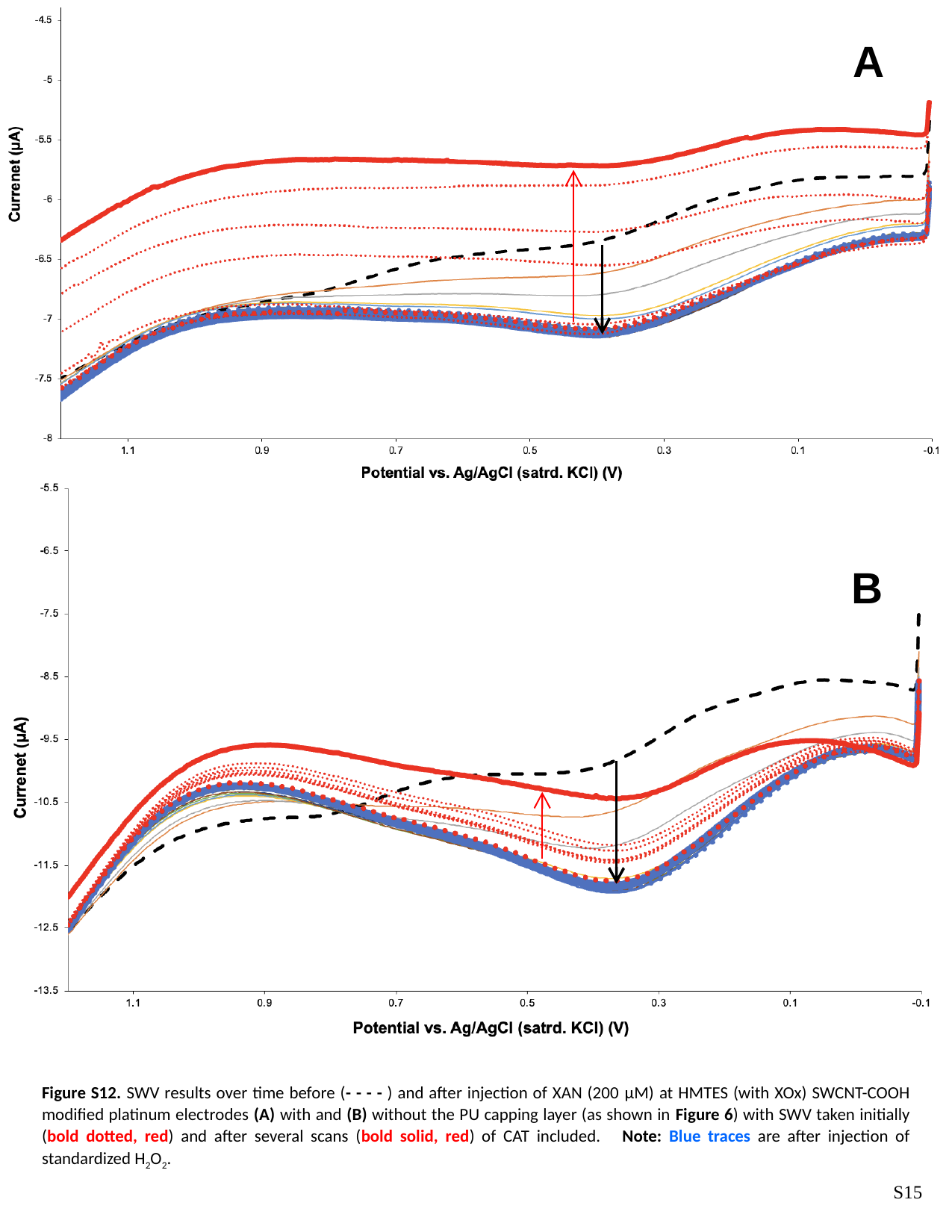

A
B
Figure S12. SWV results over time before (- - - - ) and after injection of XAN (200 µM) at HMTES (with XOx) SWCNT-COOH modified platinum electrodes (A) with and (B) without the PU capping layer (as shown in Figure 6) with SWV taken initially (bold dotted, red) and after several scans (bold solid, red) of CAT included. Note: Blue traces are after injection of standardized H2O2.
S15
